# A role for GABA in the modulation of striatal and hippocampal systems under stress

**DOI:** 10.1038/s42003-021-02535-x

**Published:** 2021-09-02

**Authors:** Nina Dolfen, Menno P. Veldman, Mareike A. Gann, Andreas von Leupoldt, Nicolaas A. J. Puts, Richard A. E. Edden, Mark Mikkelsen, Stephan Swinnen, Lars Schwabe, Geneviève Albouy, Bradley R. King

**Affiliations:** 1 Movement Control and Neuroplasticity Research Group, Department of Movement Sciences, KU Leuven, Leuven, Belgium; 2 Leuven Brain Institute, Leuven, Belgium; 3 Health Psychology, KU Leuven, Leuven, Belgium; 4 Department of Forensic and Neurodevelopmental Sciences, Institute of Psychiatry, Psychology, and Neuroscience, King’s College London, London, UK; 5 MRC Centre for Neurodevelopmental Disorders, King’s College London, London, UK; 6 Russell H. Morgan Department of Radiology and Radiological Science, The Johns Hopkins University School of Medicine, Baltimore, MD USA; 7 F. M. Kirby Research Center for Functional Brain Imaging, Kennedy Krieger Institute, Baltimore, MD USA; 8 Department of Cognitive Psychology, Institute of Psychology, University of Hamburg, Hamburg, Germany; 9 Department of Health and Kinesiology, College of Health, University of Utah, Salt Lake City, UT USA

**Keywords:** Learning and memory, Motor control

## Abstract

Previous research has demonstrated that stress modulates the competitive interaction between the hippocampus and striatum, two structures known to be critically involved in motor sequence learning. These earlier investigations, however, have largely focused on blood oxygen-level dependent (BOLD) responses. No study to date has examined the link between stress, motor learning and levels of striatal and hippocampal gamma-aminobutyric acid (GABA). This knowledge gap is surprising given the known role of GABA in neuroplasticity subserving learning and memory. The current study thus examined: a) the effects of motor learning and stress on striatal and hippocampal GABA levels; and b) how learning- and stress-induced changes in GABA relate to the neural correlates of learning. To do so, fifty-three healthy young adults were exposed to a stressful or non-stressful control intervention before motor sequence learning. Striatal and hippocampal GABA levels were assessed at baseline and post-intervention/learning using magnetic resonance spectroscopy. Regression analyses indicated that stress modulated the link between striatal GABA levels and functional plasticity in both the hippocampus and striatum during learning as measured with fMRI. This study provides evidence for a role of GABA in the stress-induced modulation of striatal and hippocampal systems.

## Abstract (web summary)

Using fMRI and MRS measurements from healthy, young adults, Dolfen et al examined the effects of motor learning and stress on striatal and hippocampal GABA levels, as well as effects on neural correlates of learning. They demonstrated that stress modulated the link between striatal GABA levels and functional plasticity in the hippocampus and striatum, which reveals a role for GABA in the stress-induced modulation of these systems.

## Introduction

There is a plethora of evidence that both the hippocampus (HC) and striatum (STR) are involved in motor sequence learning (MSL)^1–10^. During the acquisition of new movement sequences, the pattern of activation in these regions is antagonistic, such that activation increases in the STR and decreases in the HC as a function of learning (e.g., see refs. ^7,9–11^). Importantly, these particular dynamics during encoding have been linked to successful motor learning and subsequent motor memory retention^1,9,10,12^. As these neural signatures are critical for the learning and memory process, recent research has started to examine whether these brain responses can be altered through experimental interventions (e.g., see refs. ^13–16^).

An intervention that has shown promise to modify the relative engagement of hippocampal and striatal systems during learning is acute stress. Specifically, previous studies indicate that stress boosts striatal activation at the expense of hippocampal functioning during spatial navigation and probabilistic classification learning^17–21^. This shift increases striatal-dependent habitual control of learning (thought to be adaptive to deal with acute stress) but comes at the cost of flexibility of learning, supported by the HC. Recent research in our group extended these findings with evidence that stress prior to MSL favours the recruitment of motor cortical regions, known to be highly connected to the STR^22^, and results in a stronger disengagement of the hippocampal system during learning^13^. Moreover, our findings suggested that inter-subject variability in the brain responses to stress in these regions determines the impact of stress on motor learning and memory retention.

These prior studies provided critical insights into how learning and stress alter blood oxygen-level dependent (BOLD) responses in cortico-hippocampal and cortico-striatal networks. However, the exact neurochemical substrates supporting these processes are unknown. This knowledge gap is surprising given the critical role of neurometabolites in learning and memory processes. In the study of neuroplasticity associated with motor learning, the main inhibitory neurotransmitter γ-aminobutyric acid (GABA) has received considerable attention. Animal models examining the role of GABA in learning-related motor cortical plasticity indicate that long-term potentiation-like synaptic changes are associated with decreases in GABA and a release of inhibition^23,24^. In line with this, sensorimotor GABA levels in humans, measured non-invasively with magnetic resonance spectroscopy (MRS), prior to motor learning and responsiveness of motor cortical GABA to non-invasive brain stimulation have been found to be predictive of subsequent motor behaviour^25,26^. Furthermore, previous studies in humans have demonstrated decreases in sensorimotor GABA as a result of learning^27–29^. These data collectively demonstrate the importance of GABA physiology in the context of motor learning. Surprisingly, although the role of the STR and the HC in motor learning is well documented, the physiology of striatal and hippocampal GABA has never been studied in this context.

Investigations into how stress influences GABA in learning- and memory-relevant structures, including the STR and HC, are relatively scarce. A hallmark of the physical stress response is the release of corticosteroids (cortisol in humans) whose actions are influenced by mineralocorticoid and glucocorticoid receptors in the brain^30^. These receptors are expressed in all brain areas but with an increased density in the excitatory non-GABAergic principal cells of the HC. Previous work has therefore primarily focused on stress-induced modulation of non-GABAergic mechanisms in the HC (refs. ^18,19,31,32^; for reviews, see refs. ^20,33^). Accordingly, the effect of stress on functioning of GABAergic hippocampal interneurons is less understood. Nevertheless, in line with evidence of deleterious effects of stress on hippocampal functioning (see refs. ^34,35^ for reviews), previous work in rodents reported increased GABA levels in the HC after stress exposure^36^ (see ref. ^37^ for a potential mechanism of this change). Similar to the HC, the effect of stress on striatal GABA physiology remains scarcely described. The limited work in animals documented both stress-induced decreases^38–40^ as well as increases in striatal GABA^41^. In the human brain, however, studies examining stress-induced changes in GABA levels are limited to the prefrontal cortex^42,43^. Hence, reports on hippocampal and striatal regions are currently lacking.

Given that GABA plays a key role in motor learning^25,27,29^ and stress alters learning-related responses in the STR and the HC^17–21^, we investigated how stress and motor learning alter GABA levels in the HC and the STR. Moreover, we examined the relationship between GABA and BOLD signals across the STR and HC. Given that previous research assessing activity and connectivity from BOLD images has demonstrated that a competitive interaction between these two regions is crucial for motor memory processes^44^, investigations into how GABA in one region influences BOLD in the other will foster a greater understanding of the nature of this interplay during learning. In this study, participants were exposed to a stressful (Socially Evaluated Cold Pressor Test, SECPT^45,46^) or non-stressful control intervention prior to performing a MSL task while functional magnetic resonance imaging (fMRI) data were acquired. GABA levels were assessed using MRS in the HC and STR before the intervention, as well as following MSL. As acute stress has been shown to reduce hippocampal activation^13,17,47,48^, stress is hypothesized to increase GABAergic inhibition in the HC and to result in higher hippocampal GABA levels at the end of training in the stress as compared to the control group. Based on the diverse population of GABAergic neurons in the STR, striatal GABA measures presumably reflect activity of GABAergic interneurons and, when activated, e.g., during motor learning, activity of GABAergic (principal) projection neurons^49,50^. Given that stress boosts activation in motor cortical regions during MSL^13^ and these regions have massive glutamatergic projections to striatal GABAergic projection neurons^51–53^, we hypothesized stress to potentiate activity of these GABAergic neurons during learning. Therefore, we expected stress to result in increased GABA release in the STR and hence higher GABA levels post learning in the stress as compared to the control group. Last, we expected learning and stress-related changes in GABA levels to be related to task-related BOLD signals in the STR and HC (e.g., see ref. ^54^). In particular, based on the known interplay between striatal and hippocampal regions during MSL (e.g., see ref. ^1^), we predicted that GABA levels in one memory systems would relate to BOLD signal in the other memory system.

## Results

**Fig. 1:**
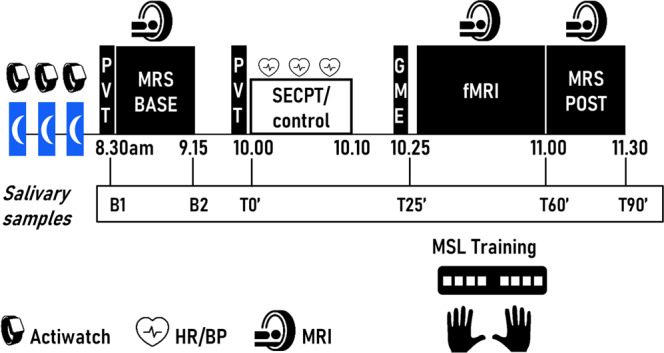
Experimental design. All participants respected a constant sleep/wake schedule for 3 nights before the experimental session. Compliance to the schedule was checked using Actigraphy. On the day of the experimental session, participants were trained on a motor sequence learning (MSL) task (bimanual finger-tapping task) during fMRI. The task was performed in a self-initiated manner and required participants to learn an eight-element sequence. Prior to MSL, subjects were randomly assigned to one of two groups according to whether they were exposed to the stress (SECPT) or control intervention. Magnetic resonance spectroscopy (MRS) data were acquired at baseline (pre-intervention; MRS base) and post intervention/learning (MRS post). Immediately before MSL, the effect of stress on general motor execution (GME) was assessed using a random serial reaction time task (see Supplementary Results). Salivary samples were collected at baseline (B1 änd B2, immediately before and after MRS base, respectively), at the start of the SECPT/control intervention (T0’), before MSL (T25’), immediately before (T60’) and after MRS post (T90’). Heart rate (HR) and blood pressure (BP) were taken before, during and after the intervention. PVT, Psychomotor Vigilance Testing; SECPT, Socially Evaluated Cold Pressor Task.

In the current study, participants were trained on a MSL task during fMRI (Fig. 1). Prior to MSL, subjects were exposed to either a stress (SECPT) or control intervention. MRS data were acquired at baseline (i.e., pre-intervention) and post intervention/learning. In line with our previous work^13,55^ and given the critical role of glucocorticoids in the impact of stress on learning and memory^20,56^, the primary group comparison presented in the main text focused on controls and stressed participants with an increase in cortisol (i.e., stress cortisol responders, SCRs) (see “Methods”).

### Effectiveness of stress induction

**Fig. 2:**
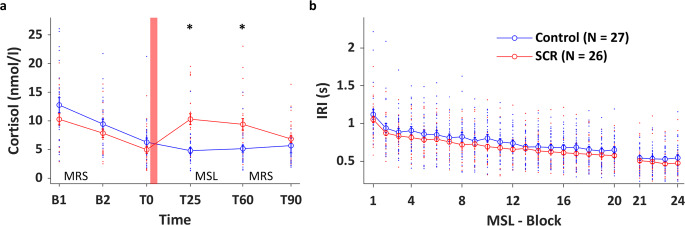
Effectiveness of the stress induction and MSL performance. Individual data plotted on top of group averages. **a** Time course of salivary cortisol concentration (nmol/L). B1 and B2, Baseline 1 and 2. MRS, MR spectroscopy. The red box represents the control/stress intervention (~T0). In the SCR group, cortisol levels were significantly elevated at the start of MSL (T25; 25 min post stress intervention) and remained elevated until 60 min post intervention (T60). Cortisol of two subjects at B1 (1 control, 1 SCR) were missing. See Supplementary Results section 2.2 (and corresponding Supplementary Fig. 2c) for results of the analysis of the time course of cortisol concentration in the stress and control groups before cortisol responder/non-responder classification. (*) Indicates significant group differences at *p*_corr_ < 0.05. **b** Performance speed (inter-response interval between consecutive correct key presses, IRI) plotted as a function of blocks of practice during MSL for the control and SCR groups. Performance speed improved with practice and to a similar rate in both groups. Error bars in all panels represent SEM.

To measure the effectiveness of the stress induction by the SECPT, subjective and physiological responses were repeatedly measured during the experiment (Fig. 1). Subjective and autonomic responses to the intervention are summarized in Table 1. With respect to the subjective response to stress, the SECPT was rated as significantly more stressful, unpleasant and painful as compared to the control manipulation (unpaired two-sample *t*-tests, Control vs. SCR, all *p*s < 0.001). Both blood pressure and heart rate significantly increased in response to the SECPT but not in response to the control intervention [3 (Time: pre vs. during vs. post) × 2 (Groups) repeated-measures (RM) analyses of variance (ANOVAs); Time × Group interaction: all *F*s ≥ 8.573, *η*_p_^2^ ≥ 0.144, all *p*s < 0.001; see Table 1 for between-group comparisons for each time point). Finally, a 6 (Time) × 2 (Groups) RM ANOVA on cortisol concentration (nmol/l) revealed a significant Time × Group interaction [*F*_(2.303, 112.865)_ = 12.239, *η*_p_^2^ = 0.153, *p* < 0.001]. As shown in Fig. 2a, the SECPT triggered an endocrine response, which resulted in significantly elevated cortisol concentrations in the SCR as compared to the control group at T25’ (*p* < 0.001) and T60’ (*p* < 0.001). As the cortisol concentration in controls was higher at the start of the baseline MRS measurements (B1) as compared to MRS post intervention/learning (T60) (*p* = 0.009) (Fig. 2a), we performed correlational analyses to investigate the link between cortisol concentrations at the start of each MRS time point and corresponding GABA+ measures. These follow-up analyses revealed no significant correlations (all *p*s_uncorr_ ≥ 0.20).

**Table 1:** Subjective and autonomic (heart rate, systolic and diastolic blood pressure) responses to the stressor.

*Subjective ratings*^a^			
	Pain	Stress	Unpleasantness
Control	1.39 ± 4.79	1.26 ± 4.34	1.1 ± 4.33
SCR	68.15 ± 21.07	55.47 ± 24.9	88.87 ± 12.13
Control vs. SCR	*p* *<* 0.001	*p* *<* 0.001	*p* *<* .001
*Autonomic responses*^b^			
	Pre	During	Post
SBP (mm Hg)			
Control	122.19 ± 10.45	120.85 ± 9.50	120.37 ± 10.05
SCR	130.00 ± 13.33	144.54 ± 20.38	133.19 ± 13.63
Control vs. SCR	*p* *=* 0.021	*p* *<* 0.001	*p* *<* 0.001
DBP (mm Hg)			
Control	74.67 ± 6.65	73.96 ± 5.81	74.07 ± 5.59
SCR	75.08 ± 9.16	90.92 ± 17.65	83.54 ± 14.76
Control vs. SCR	*p* *=* 0.852	*p* *<* 0.001	*p* *=* 0.003
HR (bpm)			
Control	64.48 ± 8.79	66.44 ± 9.05	68.19 ± 9.23
SCR	63.42 ± 8.87	87.65 ± 17.10	71.27 ± 11.82
Control vs. SCR	*p* *=* 0.664	*p* *<* 0.001	*p* *=* 0.294

Values are means ± SDs. *N* control group = 27; *N* SCR group = 26. Subjective ratings were given on a 100 mm visual analogue scale.

*Bpm* beats per minute, *DBP* diastolic blood pressure, *HR* heart rate, *SBP* systolic blood pressure, *SCR* stress cortisol responders.

^a^*p*-Values based on unpaired two-sample *t*-tests.

^b^*p*-Values based on pairwise comparisons following RM ANOVAs with Bonferroni correction for multiple comparisons. It is noteworthy that during the SECPT, the measurement pre-feet submersion is taken within the stressful context (including video monitoring), which likely contributed to the group difference in SBP observed at baseline.

### Performance on the MSL task

MSL consisted of 20 blocks of practice followed by an immediate post test (after a 2 min break) of 4 practice blocks, in order to minimize the confounding effect of fatigue on end-training performance^57^. Motor performance was measured in terms of speed (mean inter-response interval between two consecutive correct key presses in s) and accuracy (% of correct transitions). It is noteworthy that as performance accuracy remained stable with low error rates (see Supplementary Results section 2.5), the analyses presented in the main text focused on performance speed. A 20 (Blocks of practice) × 2 (Groups. SCR vs. Control) RM ANOVA on performance speed during training revealed that participants became faster with practice [Block: *F*_(4.269, 217.735)_ = 61.117, *η*_p_^2^ = 0.545, *p* < 0.001]. Performance improvement was not statistically different between groups [Group: *F*_(1,51)_ = 1.399, *η*_p_^2^ = 0.027, *p* = 0.242; Block × Group: *F*_(4.269, 217.735)_ = 0.566, *η*_p_^2^ = 0.011, *p* = 0.699] (Fig. 2b). Similar results were obtained for the immediate post test [4 × 2 RM ANOVA; Block: *F*_(3, 153)_ = 2.944, *η*_p_^2^ = 0.055, *p* = 0.035; Group: *F*_(1,51)_ = 0.817, *η*_p_^2^ = 0.016, *p* = 0.370; Block × Group: *F*_(3,153)_ = 1.699, *η*_p_^2^ = 0.032, *p* = 0.170]. In summary, and consistent with our previous work^13,55^, stress did not modulate performance during MSL.

### MRS of GABA

**Fig. 3:**
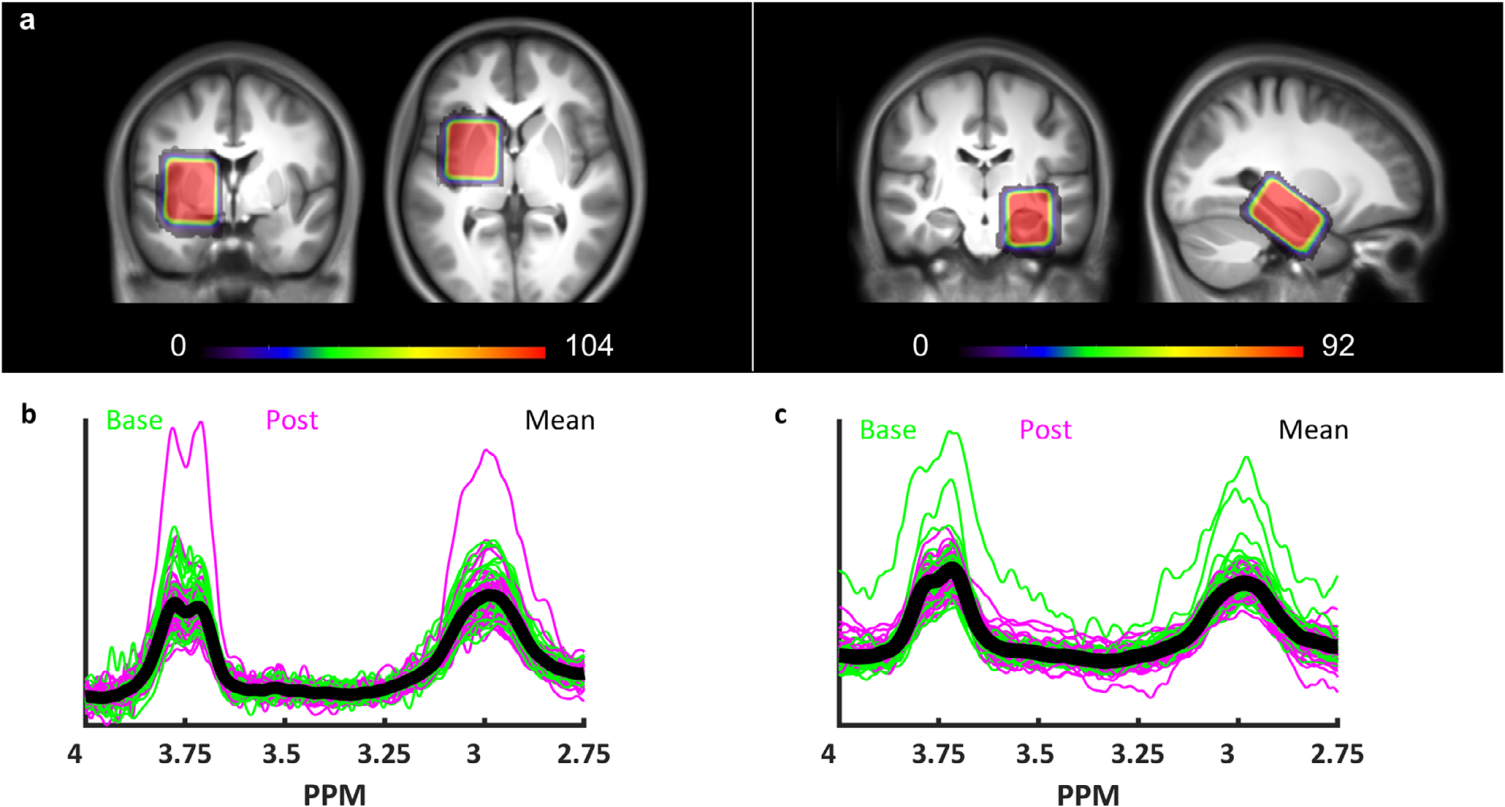
MRS. **a** Heatmaps representing the spatial overlap across participants and time points (baseline and post) for the STR (left panel) and HC (right panel) MRS voxels (STR: *N* = 104 voxels; HC: *N* = 92 voxels). Colour bars represent the number of overlapping voxels. Heatmaps are overlaid over the mean structural image across all participants (STR: *N* = 52 subjects; HC: *N* = 46 subjects). The high degree of spatial overlap indicates that there was a high consistency in voxel placement across time points and individuals for both voxels. **b** Spectra of all STR MRS measurements and **c** HC measurements from all participants and time points. GABA+ peak is visible at 3 p.p.m. Baseline and post intervention/learning time points are depicted in green and magenta, respectively (mean spectrum across all participants and time points depicted in black). It is noteworthy that although there are extreme peaks, none of the extracted GABA+ levels were statistical outliers. Base, baseline pre-intervention/learning. Post, post intervention/learning.

GABA-edited MRS of the left STR and the left HC was performed before the intervention (baseline) and immediately after MSL (post intervention/learning) (Fig. 1). Data quality metrics and MRS voxel tissue fractions for each region of interest and corresponding analyses are detailed in the Supplementary Results (Section 2.6 and Supplementary Table 3). The consistency of voxel placement was high for both regions, as shown by the heatmaps in Fig. 3a that depict spatial overlap of the MRS voxels. STR and HC MRS spectra are depicted in Fig. 3b, c.

**Fig. 4:**
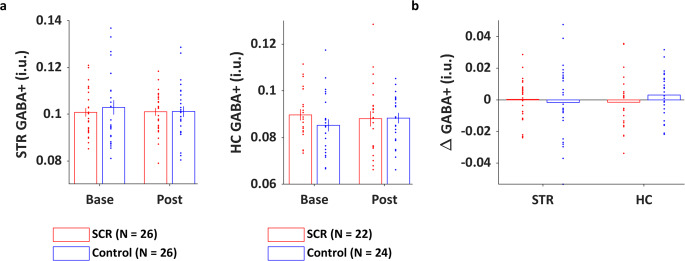
GABA+ levels and GABA+ changes. **a** Individual GABA+ levels for each time point plotted on top of group average for the SCR and control groups. There were no effects of time, group or any group by time interactions. Error bars reflect SEM. **b** Individual GABA+ changes (ΔGABA+) plotted on top of group average. These values were used in regression analyses to assess the link between GABA+ and both motor performance and BOLD. Consistent with the analyses of the data depicted in **a**, there were no differences in ΔGABA+ between groups (unpaired two-sample *t*-tests, all *p*s > 0.1). Positive values reflect increases, negative values reflect decreases. Base, baseline pre-intervention/learning. Post, post intervention/learning.

To investigate the effect of stress/learning on GABA+ levels, for each region, a 2 (Time: baseline vs. post) × 2 (Groups: SCR vs. Control) RM ANOVA was conducted. There was no significant effect of group or time on STR GABA+ levels, neither was there a time × group interaction (Fig. 4a, left panel) [Time: *F*_(1,50)_ = 0.088, *η*_p_^2^ < 0.001, *p* = 0.768; Group: *F*_(1,50)_ = 0.144, *η*_p_^2^ < 0.001, *p* = 0.563; Time × Group: *F*_(1,50)_ = 0.144, *η*_p_^2^ < 0.001, *p* = 0.706]. Similarly, the 2 × 2 RM ANOVA performed on HC GABA+ yielded no significant effects [Time: *F*_(1,44)_ = 0.089, *η*_p_^2^ = 0.002, *p* = 0.766; Group: *F*_(1,44)_ = 0.524, *η*_p_^2^ = 0.012, *p* = 0.473; Time × Group: *F*_(1,44)_ = 0.974, *η*_p_^2^ = 0.034, *p* = 0.329] (see Fig. 4a, right panel). Altogether, these results indicate that, at the group level, neither stress (prior to MSL) nor MSL influenced GABA+ levels in the STR and the HC.

### BOLD responses during the MSL task

**Fig. 5:**
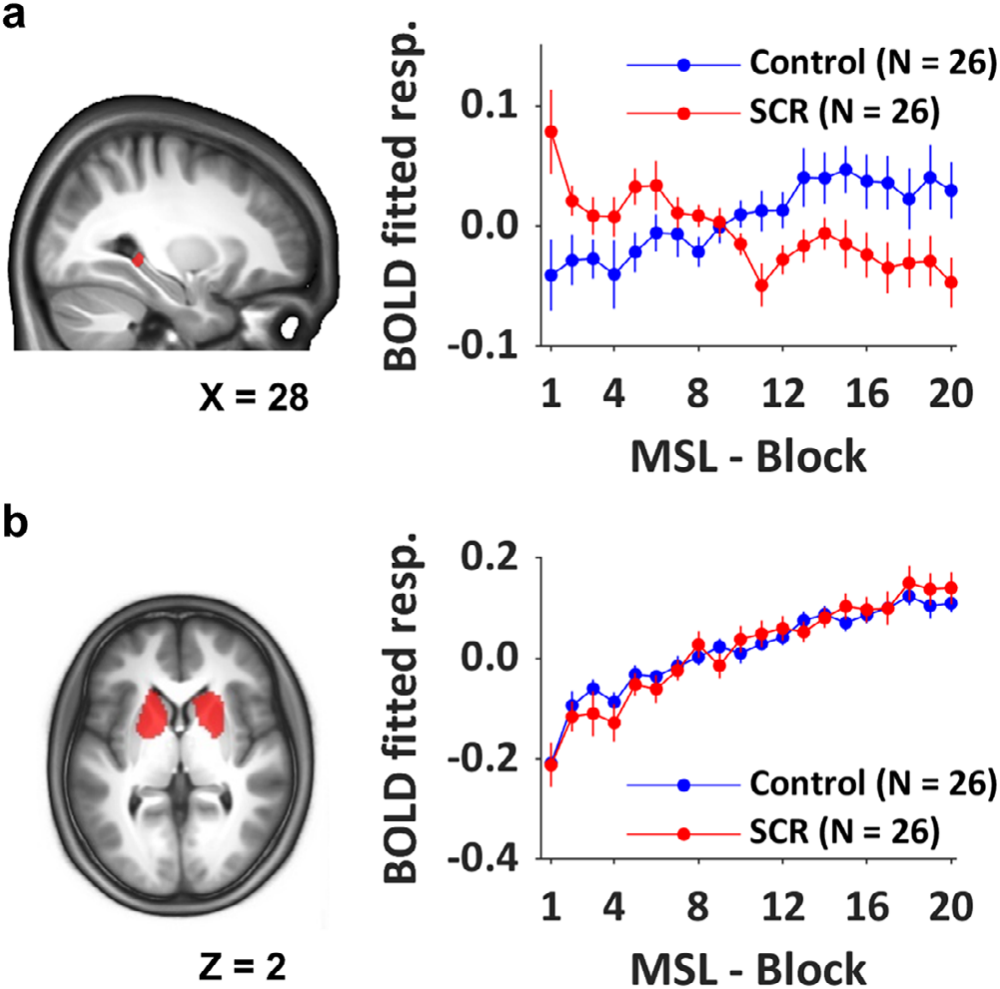
Linear modulation of brain responses by performance speed. To show the modulation effect across blocks of practice, the β-estimate was multiplied with the modulation regressor vector and data were then averaged across time points within each block of practice. It is noteworthy that due to missing fMRI data, one extra control participant was excluded. **a** Effect of stress on HC dynamical activation ([30 −36 −2mm], 69 voxels, Control vs. SCR, *T* = 3.05, *p*_SVC_ = 0.036). The averaged BOLD fitted response shows that right HC activation decreased more across blocks of practice in the stress group as compared to controls. **b** Across groups, bilateral STR responses increased with performance speed improvement. For illustrative purposes, the graph depicts the groups separately and the averaged BOLD fitted response is based on the right dorsal putamen ([22 16 2 mm]) but other clusters exhibited similar results (see Supplementary Table 5 for all clusters). Error bars indicate SEM in both panels. For display purposes, activation maps are overlaid on a T1-weighted template image with a threshold of *p* < 0.005 uncorrected.

A primary objective of the current research was to relate the STR/HC GABA+ measures to BOLD responses during MSL. Before presenting these data, we report here patterns of task-related brain activation in our regions of interest (ROI) during MSL, independent of the relationship to GABA+. ROIs included bilateral hippocampi and bilateral STR (caudate nuclei and putamen). Corresponding results from the full sample and performed on the whole brain are described in Dolfen et al.^13^. Contrasts of interest were the main effect of practice and its linear modulation by performance speed during MSL practice. Modulation contrasts identify regions where the magnitude of the BOLD responses increased (or decreased) as speed of performance increases over the course of practice. Results did not show any significant differences in brain activation, in the ROIs between SCR and controls for the main effect of task practice. However, stress altered the pattern of dynamical activation in hippocampal regions during practice (i.e., modulation of brain activation by performance). Specifically, in stressed participants, the right hippocampal activation decreased significantly more in proportion to performance speed as compared to controls ([SCR-Control], (30 -36 -2 mm), *T* = 3.05, *p*_svc_ = 0.036) (Fig. 5a). Stress did not modulate striatal dynamical patterns: activation significantly increased as a function of practice in both groups (Fig. 5b and see Supplementary Table 5).

### Link between GABA+ and BOLD responses during MSL

We investigated the link between STR/HC GABA+ measures and BOLD responses in our ROIs and, in particular, whether this link was modulated by stress. To do so, we conducted fMRI regression analyses using either baseline GABA+ or ΔGABA+ (i.e., ΔGABA+; computed as raw change in GABA+ from baseline to post intervention/learning; see Fig. 4b) as covariates. For each contrast of interest, we describe any group differences in the relationship between brain activation and STR/HC GABA+ measures. Results of these between-group comparisons are summarized in Table 2 and follow-up within-group regression results are presented in Supplementary Table 6.

**Table 2:** Neuroimaging regression analyses: results of between-group comparisons.

Region	*X* mm	*Y* mm	*Z* mm	# Voxels	*T*	*p*
*Regression with STR baseline GABA+*						
Main effect of practice						
[Control-SCR]						
No suprathreshold clusters						
[SCR-Control]						
Hippocampus	−18	−24	−10	31	3.13	0.031
Modulation by speed of performance						
No suprathreshold clusters						
*Regression with HC baseline GABA+*						
Main effect of practice						
[Control-SCR]						
No suprathreshold clusters						
[SCR-Control]						
Hippocampus	28	−36	0	30	2.98	0.031
Modulation by speed of performance						
No suprathreshold clusters						
*Regression with ΔGABA+ STR*						
Main effect of practice						
[Control-SCR]						
Hippocampus	−20	−14	−20	79	3.42	0.015
[SCR-Control]						
No suprathreshold clusters						
Modulation by speed of performance						
[Control-SCR]						
No suprathreshold clusters						
[SCR-Control]						
Putamen	20	14	6	218	3.39	0.016
(extending to caudate)						
*Regression with ΔGABA+ HC*						
No suprathreshold clusters						

Significance level set at *p*_corr_ < 0.05 corrected for multiple comparisons (FWE) over small volumes. All results survived Holm–Bonferroni correction for multiple testing. Due to missing fMRI data, one control participant was not included in the regressions with HC GABA+: *N* control = 23, *N* SCR = 22. Regressions with STR GABA+: *N* control = 26, *N* SCR = 26.

*ΔGABA+* post intervention/learning minus baseline, *HC* hippocampus, *STR* striatum, *SCR* stress cortisol responders.

#### Baseline GABA+ and BOLD responses

**Fig. 6:**
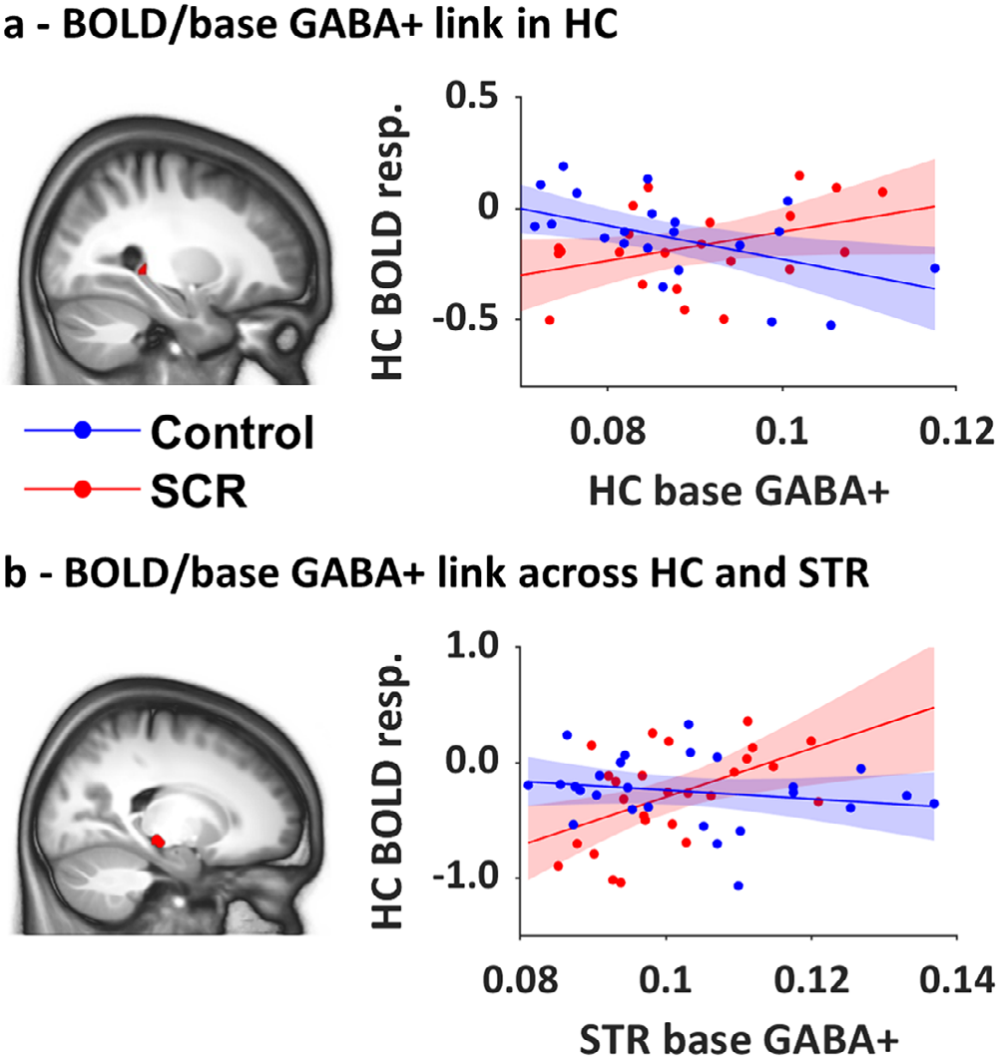
Correlations between task-related BOLD responses during MSL and baseline GABA+ measures. For display purposes, activation maps are overlaid on a T1-weighted template image with a threshold of *p* < 0.005 uncorrected. Regression plots represent BOLD responses (resp.) [practice > rest] against baseline GABA+ measures for the two groups. **a** Link between BOLD and GABA+ within the HC: right HC activation [28 −36 0 mm] was differently correlated with HC baseline GABA+ in the SCR and control groups. **b** Link between BOLD and GABA+ across regions: left  HC activation [−18 −24 −10 mm] was differently correlated with STR baseline GABA+ in the SCR and control groups. Due to missing fMRI data, one control participant was not included in the regressions with HC GABA+: *N* Control = 23, *N* SCR = 22. Regressions with STR GABA+: *N* Control = 26, *N* SCR = 26. BOLD and GABA measures are in arbitrary units.

We first correlated baseline HC/STR GABA+ levels to the brain activation maps of the main effect of practice. The regression analysis with HC baseline GABA+ showed that stress modulated the link between HC baseline GABA+ levels and right hippocampal BOLD activation during task practice (Fig. 6a). Within-group, follow-up analyses revealed that HC baseline GABA+ was negatively related to hippocampal activation in the control group, i.e., lower HC GABA+ levels were associated with overall higher hippocampal activation, but not in the stress group. A group difference was also found in the link between STR baseline GABA+ and left hippocampal activation (Fig. 6b). Specifically, in the SCR but not in the control group, there was a significant positive relationship between STR baseline GABA+ and left hippocampal activation, such that lower STR baseline GABA+ was associated with overall less hippocampal activation. Next, we correlated baseline GABA+ measures with the individual brain activation maps of modulation by performance. No group differences in the link between HC/STR baseline GABA+ and BOLD modulation were observed in our ROIs.

#### ΔGABA+ and BOLD responses

**Fig. 7:**
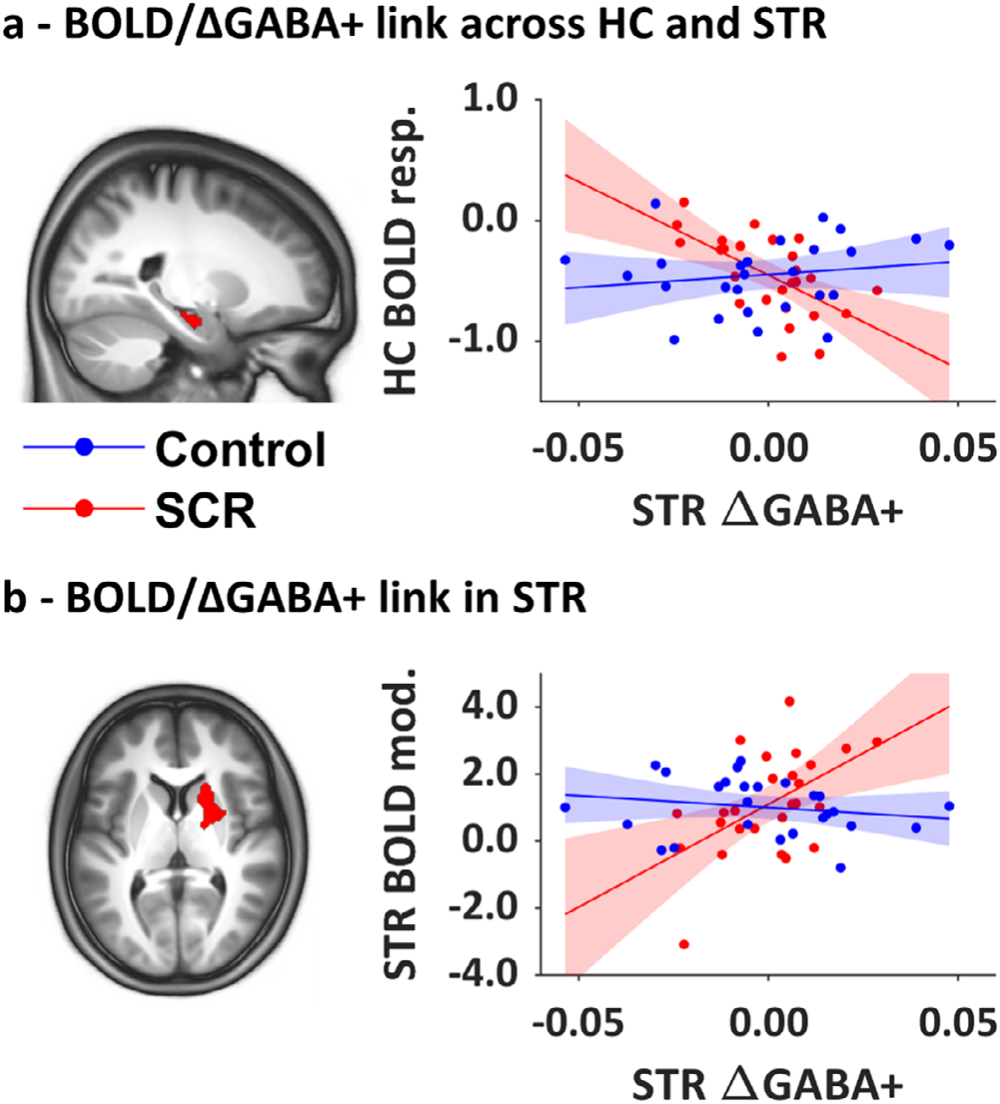
Correlations between task-related BOLD responses during MSL and striatal (STR) ΔGABA+. For display purposes, activation maps are overlaid on a T1-weighted template image with a threshold of *p* < 0.005 uncorrected. Regression plots represent BOLD responses (resp.) [practice > rest] (**a**) or magnitude of practice-related modulation (mod.) in BOLD responses (**b**) against STR ΔGABA+ for the two groups. **a** Link between ΔGABA+ and BOLD across regions: brain responses in the left HC [−20 −14 −20 mm] were differently correlated with changes in STR GABA+ (ΔGABA+; i.e., post minus baseline) in the SCR and control groups. **b** Link between ΔGABA+ and BOLD within the STR: dynamical brain responses in the right putamen [20 14 6 mm] were differently correlated with ΔGABA+ in the SCR and control groups. A positive BOLD modulation value reflects an increase in activation over learning. Regressions with STR ΔGABA+: *N* Control = 26, *N* SCR = 26. BOLD and GABA measures are in arbitrary units.

Similarly, individual brain activation maps of the main effect of practice were correlated to STR/HC ΔGABA+. The regression with STR ΔGABA+ showed that stress modulated the link between STR ΔGABA+ and left hippocampal activation during MSL (Fig. 7a). Specifically, a larger decrease in STR GABA+ over training in stressed participants was associated with overall higher activation in the left HC. To ensure that our results were specific to GABA+ changes, a partial correlation was computed in the SCR group between STR ΔGABA+ and HC BOLD, while controlling for STR baseline GABA+. Importantly, the relationship in the SCR group remained significant after controlling for STR baseline GABA+ (*p* = 0.042).

With respect to the link between magnitude of BOLD modulation by performance and ΔGABA+, regression analyses with STR ΔGABA+ yielded group differences in a right striatal cluster including the putamen and caudate (Fig. 7b). In line with our hypotheses, the findings indicated that practice-related increases in STR BOLD during MSL are linked to increases in STR GABA+ from baseline to post in stressed participants. It is noteworthy that the link between STR ΔGABA+ and STR BOLD modulation in the SCR group remained significant when controlling for STR baseline GABA+ (*p* = 0.025). No group differences in the link between HC ΔGABA+ and brain responses in our ROIs were observed.

## Discussion

In the current study, we investigated the effects of stress and motor learning on the levels of striatal and hippocampal GABA, and how they relate to BOLD responses in these regions during MSL. Our results indicate that neither stress nor subsequent learning influenced GABA+ at the group level. Importantly, however, stress altered the link between STR GABA+ levels and task-related activation in the HC. These results provide evidence for a link between GABA and BOLD signals across the HC and STR, and ultimately support the view that GABA plays a role in the modulation of striatal and hippocampal systems under stress.

### Stress modulates the link between baseline inhibitory tone and BOLD responses

Our data indicated that the two groups differed with respect to the relationship between baseline hippocampal GABA levels and task-related functional activation in the HC. The role of the HC in MSL is well documented. In line with evidence of hippocampal involvement in spatial information processing (e.g., see refs. ^58,59^) and associating sequential events (e.g., see ref. ^60^), hippocampal activation during MSL is described to support the sequential and abstract nature of the to-be-learned material. Furthermore, the HC is crucial for the development of an abstract allocentric representation of the movement sequence and is thought to trigger sleep-dependent memory consolidation processes^1^. Although previous studies underscore the importance of hippocampal recruitment during motor memory encoding^1,9,10,12^, the neurochemical mechanisms supporting this remain to be explored in humans. Our findings suggest that low baseline inhibitory tone in the HC, presumably reflecting decreased GABAergic interneuron activity^61^, was predictive of larger task-related hippocampal activation in the control group. The direction of the relationship is in accordance with a recent study showing a similar inverse link between hippocampal GABA and hippocampal BOLD magnitude during thought suppression^62^. Importantly, the current study showed that stress disrupted this link, suggesting that inter-subject variability in hippocampal baseline inhibitory tone does not relate to hippocampal functional (BOLD) plasticity under stress.

Interestingly, inter-subject variability in striatal baseline inhibitory tone accounted for differences in the magnitude of hippocampal BOLD responses in the stress but not the control group. Analogous to the HC, baseline striatal GABA levels in this study might reflect striatal GABAergic interneuron activity and, therefore, higher striatal GABA levels can be interpreted as greater GABAergic inhibition on striatal principal neurons^63^. Importantly, the observation that higher baseline striatal inhibitory tone is related to more hippocampal BOLD activation in stressed participants is in line with the known competitive interplay between striatal and hippocampal systems^64^. When considering previous evidence of stress-induced decreases in hippocampal functional activation in the same time window^17,18,32,65^, it’s tempting to speculate that a high baseline striatal inhibitory tone might protect the HC from negative effects of stress. Alternatively, it might suggest that the high striatal inhibitory tone prevents the known stress-induced shift from hippocampal-dependent towards striatal-dependent control over learning^17,18^ and results in the maintenance of hippocampal activation. It is noteworthy that such interpretations are certainly speculative and we cannot differentiate between these possibilities. Importantly, our results not only show that stress promoted the link between striatal GABA tone and hippocampal responses but also that stress disrupted the link between hippocampal inhibitory tone and hippocampal functional activity. Altogether, these findings suggest a potential role for striatal GABA in hippocampal functional plasticity under stress.

### Stress modulates the link between GABA changes and BOLD responses

Under stress, as compared to control conditions, greater learning-related increases in striatal activation were associated with a net increase in striatal GABA levels. Although inferences about neural mechanisms are relatively limited based on MRS, this pattern of results potentially suggests that our post GABA measurement in the STR might be driven by changes in GABA release from projection neurons. This possibility is indeed highly likely when considering the local neuronal population in the STR that encompasses both GABAergic inhibitory interneurons and GABA releasing principal neurons. The small group of GABAergic interneurons exert control over the principal GABAergic projection neurons, which account for 74% of the striatal neuronal population^66^. Importantly, previous work in rodents has shown that these striatal GABAergic projection neurons are, in contrast to the striatal inhibitory interneurons, largely inactive at rest and need glutamatergic cortical inputs to discharge^52,53^. These observations confirm that the baseline striatal GABA levels discussed above might indeed mainly reflect interneuron activity. They also suggest that experimental interventions such as learning/stress might activate the large population of striatal GABAergic projection neurons and therefore induce GABA release. When considering the local neuronal population in the STR, the possibility seems indeed likely that increased striatal activation is paralleled by increased activation of GABAergic projection neurons and thus increases in striatal GABA. Although we did not observe an intervention-related increase in striatal GABA at the group level, the significant positive relationship between net GABA changes and striatal activation found in our stress group is in line with this interpretation. Moreover, stress also modulated the link between changes in striatal GABA levels and hippocampal BOLD responses during task practice. Our results then collectively revealed that, an intervention-related increase in striatal GABA in the stress group is correlated with larger learning-related increases in striatal activation and overall lower hippocampal activation. These results further highlight a stress-induced competition between striatal and hippocampal systems across GABA and BOLD signals. In particular, they are in line with previous literature on the effect of stress on striatal-hippocampal interactions (for a review, see ref. ^34^).

### Stress and the interplay between hippocampal and striatal systems

Accumulating evidence suggests that stress promotes striatal-related behaviour (also called ‘rigid’) at the expense of more ‘flexible’ behaviour supported by the HC (for a review, see ref. ^67^). This shift has extensively been studied within the context of spatial navigation and procedural classification learning. In both types of learning, the task can be solved by using flexible spatial or allocentric strategies (based on multiple cues/implicit cue patterns) and rigid stimulus-response (S-R) or egocentric learning strategies (based on explicit single cues)^67^. At the neural level, learning in these tasks has consistently been shown to rely on multiple memory systems. Specifically, the use of rigid S-R strategies depends on the striatal-based procedural memory system^68^, whereas more spatial or allocentric strategies require the hippocampal-based declarative memory system^58,69^. fMRI studies show that the stress-induced shift in strategy on the behavioural level is paralleled by a shift at the neural level. Specifically, stress results in decreased hippocampal activation^17,18,32,65^ and increased striatal activation^70^. In the current study, we extend these previous findings and showed that GABA levels in the STR are linked to both hippocampal and striatal functional activation under stress. Moreover, in line with evidence of inhibitory connections between these areas, whereby inactivation of the HC enhanced STR-dependent learning^71,72^ and disruption of the STR facilitates HC-dependent learning^73^, we observed a similar competitive interaction pattern across BOLD and GABA signals under stress. Evidence that stress affects the relative involvement of striatal and hippocampal networks in learning and favours striatal-dependent processes at the expense of hippocampal-dependent processes (see ref. ^34^ for a review) further supports such an interaction in the stress rather than in the control group.

### Stress, GABA and motor learning

Contrary to our expectations, stress and learning did not induce changes in striatal or hippocampal GABA at the group level. It is worth noting that the lack of a significant effect is in contrast to what has previously been observed in the primary motor cortex (M1). Indeed, MRS studies have provided evidence of disinhibition in sensorimotor regions (i.e., decreases in sensorimotor GABA levels) over the course of motor learning, and MSL in particular^27,28^. The decrease in inhibition has been described to facilitate learning-related plasticity^25^. Given the direct glutamatergic projections from M1 to the STR^22^, one could also have expected to observe learning-related changes in GABA downstream in striatal regions. Although BOLD responses increased in cortico-striatal networks as a function of the robust behavioural improvements, we found no evidence that the learning episode modulated striatal GABA at the group level. Similarly, although our previous work showed that stress induced a boost in activation in motor cortical areas^13^ and striatal-dependent processes are typically facilitated by stress (e.g., see ref. ^70^), the administration of stress prior to learning in this study also unexpectedly did not influence striatal GABA levels.

With respect to the HC, fMRI investigations have shown that the HC is primarily active early during MSL and its activation gradually decreases thereafter^7,9–11^. Moreover, our previous work indicated that stress resulted in a larger practice-related decrease of hippocampal BOLD responses during learning^13^. These changes in functional activation were not paralleled by changes in hippocampal GABA levels. Although previous literature examining the interaction among GABA, stress and learning in the motor domain is limited, insights can be gleaned from the declarative memory domain. Specifically, the relationship between hippocampal GABA levels and learning processes has recently been studied in associative learning^74^. In line with our study, the authors did not observe a change in hippocampal GABA over learning (note that in contrast to our study, MRS was assessed after multiple training sessions over a period of weeks); however, baseline GABA was tightly linked to memory performance.

Our findings raise the possibility that functional plasticity (induced by the stress intervention and/or motor learning) is not paralleled by GABAergic modulation in the STR and the HC. This explanation is certainly possible, yet there are also other factors that might contribute to the null effects at the group level reported here. First, due to higher field inhomogeneity (caused for example by the neighbouring ventricles), signal-to-noise ratio in subcortical regions such as the STR and the HC is lower than in the cortex^75^. Hence, changes in GABA are overall harder to detect and might be smaller than in motor cortical regions. Second, to obtain sufficient signal-to-noise ratio, the MRS voxel is rather large and, as a result, includes not solely the specific ROI. The consequences of this are twofold: (1) inter-subject variability in the size of different structures might contribute to variability in the anatomical composition of the voxel and (2) differences in physiological properties of the included structures (e.g., relative density of glutamatergic vs. GABAergic receptors) will influence estimates of GABA levels averaged across the entire voxel. Nonetheless, our regression results provide a link between inter-subject variability in GABA measures and functional plasticity during MSL.

### Limitations and future directions

MRS measures of GABA combine a mixture of GABA pools (pre-synaptic, synaptic and extracellular) from all types of GABAergic neurons in the MRS voxel. Accordingly, MRS does not allow us to draw conclusions on the exact underlying molecular mechanisms. In addition to the methodological considerations, this work provided no evidence of a link between GABA measures and behaviour. Therefore, this work is inconclusive on the behavioural relevance of GABA levels. Furthermore, to conclude whether striatal and hippocampal activation under stress truly depends on GABAergic inhibition in the STR, experimental manipulations of GABA are required rather than the individual-differences regression approach used here. It is also worth noting that BOLD data were acquired during MSL, whereas GABA was assessed pre-stress and post learning. Accordingly, our post-learning MRS time point was not acquired during the peak stress response (see Fig. 2a). It is unclear whether the relationships between GABA and BOLD signals reported in the current manuscript would remain if data were acquired simultaneously. Follow-up studies should consider using online functional MRS (e.g., see refs. ^27,76^) rather than a single post-learning measurement as used here, allowing a more temporally, fine-grained assessment.

Given our research aims, we used an MRS sequence optimized for the detection of GABA (i.e., Mescher–Garwood point-resolved spectroscopy (MEGA-PRESS)), which comes at the cost of the accurate measurement of other metabolites^77^. Interestingly, when considering modulation of other neurochemicals during learning, a recent study provided the first evidence of selective glutamate modulation in the HC during associative learning^78^. In the study of Stanley et al.^78^, the modulation of glutamate was limited to the first epochs of memory encoding. These effects are not only consistent with evidence from lesion and molecular studies for the relevance of the early hippocampal involvement in memory formation^79,80^, but also with studies in MSL, suggesting that early hippocampal activation is critical to tag the memory trace for sleep-dependent memory consolidation^1^. It is noteworthy that the MEGA-PRESS sequence used in the current study allows to quantify Glx (a combined measure of glutamate and glutamine) but these measures are thought to present low sensitivity to glutamate changes (due to high glutamine levels in the voxel)^81^. Therefore, future studies using glutamate-specific acquisition sequences should investigate MSL-related modulation of this particular metabolite of interest.

## Conclusion

This is the first study, to our knowledge, which examined the effects of stress and learning on the levels of striatal and hippocampal GABA, two structures known to be critically involved in MSL^1,4^. Our results demonstrate that neither stress nor subsequent learning had an effect on GABA at the group level. However, stress modulated the relationship between striatal GABA and hippocampal activation during motor sequence memory acquisition, providing evidence for a GABA-related link between striatal and hippocampal memory systems under stress.

## Material and methods

The research presented in this study is part of a larger experimental protocol completed by the same cohort of participants (see Supplementary Fig. 1 for the full design). A subset of corresponding results has been published in Dolfen et al.^13^.

### Participants

Eighty young (mean age: 22.2, range: 18–31, 48 females), right-handed (Edinburgh Handedness Inventory^82^), healthy individuals participated in this research. Participants had no history of neurological or psychiatric diseases and were free of medications. Participants presented no signs of chronic pain (Pain Catastrophizing Scale^83^), extreme stress (Perceived Stress Scale^84^), excessive daytime sleepiness (Epworth Sleepiness Scale^85^), anxiety (Beck Anxiety Inventory^86^) or depression (Beck Depression Inventory^87^). All participants reported normal sleep quality and quantity during the month and the night prior to the study, as evaluated with the Pittsburgh Sleep Quality Index^88^ and the St Mary’s Hospital questionnaire^89^, respectively. We did not include extreme morning or evening chronotypes (Circadian Rhythm questionnaire^90^), or shift workers. All participants provided written informed consent before the start of the study, in accordance with the local ethics committee approval (Medical Ethics Committee University Hospital Leuven, Belgium; B322201525025).

As glucocorticoids play a critical role in the impact of stress on learning and memory^20,56^, and earlier studies have shown that not all individuals show a cortisol response to the SECPT intervention (i.e., SCRs)^46^, individual cortisol data were analysed during collection. SCRs are defined as participants with a stress-induced increase larger than 15.5% or 1.5 nmol/l^91^ (classification used in refs. ^13,55^). Data acquisition continued until the number of SCRs (and control participants) reached the estimated sample size. Accordingly, 34 (21 females; 62%) and 46 (27 females; 59%) participants were subjected to the control and stress intervention, respectively. In the stress group, 17 were classified as stress cortisol non-responders (SCNR) and 29 participants as SCR. Four participants in the control group were excluded, because they were classified (using the criterion mentioned above) as cortisol responders. Two participants (one control and one SCR) were discarded, because they were statistical outliers (average ± 3 SDs) in performance speed and accuracy at the immediate post-training test. One control participant was excluded due to excessive motion during the fMRI training session (>2 voxels) and one participant (SCR group) was excluded because of a deviation from the experimental protocol. Three additional participants (one in each group) were excluded due to missing MRS data at one of the time points. Accordingly, a total of 69 participants were included in the behavioural and stress physiology analyses [Control group (*N* = 27, 16 females); Stress group (*N* = 42)]. In line with our previous work, the primary group comparison presented in the main text focused on the controls (*N* = 27, 16 females) and cortisol responders in the stress group (*N* = 26, 13 females). For completeness, all results from the relatively small set of stress cortisol non-responders (*N* = 16, 13 females) are detailed in the Supplementary Results. Participant characteristics for each of the three groups can be found in Supplementary Table 1. As the missing MRS data resulted in a slightly different sub-sample of participants as compared to Dolfen et al.^13^, results from the behavioural and stress physiology analyses from the current sample are presented in the results section but are fully in line with those presented in Dolfen et al.^13^.

Additional participants were excluded from region-specific MRS analyses and corresponding regression analyses with behaviour and brain activation. With respect to the STR, one participant (Control group) with extreme baseline GABA+ values (average + 3 SDs) was excluded. In the end, a total of 52 striatal voxels were included for each time point (corresponding to 26 control (15 females) and 26 SCR (13 females)). With respect to MRS data quality in the HC, five participants were excluded due to spectral artefacts at one of the time points resulting in no discernible GABA peak (three in the Control group, two in the SCR group). One outlier in GABA+ levels (average + 3 SDs) was identified at baseline and post intervention (both SCR group). Thus, a total of 46 hippocampal voxels were included for each time point (24 control (13 females) and 22 SCR (12 females)).

### Experimental procedure

The experimental procedure is depicted in Fig. 1. Participants followed a regular sleep/wake schedule (according to their own schedule ±1 h) starting 3 days before the experimental session. Sleep diaries and wrist actigraphy (ActiGraph wGT3X-BT, Pensacola, FL) were used to assess compliance to this schedule. On the day of the experimental session, participants were instructed to wake up at the latest 1 h before the start of the experimental session to account for the cortisol awakening response^92^. They were also not allowed to brush their teeth, eat and drink (apart from water) for 1 h before the start of the experiment, to guarantee adequate saliva sampling for cortisol assessment (see below).

At ~8.30 a.m., participants were positioned in the MR scanner for the first MRI session. During this session, baseline (i.e., pre-intervention/learning) MRS data were acquired (MRS base, Fig. 1). After this baseline session, participants were randomly assigned to one of two groups according to whether they were exposed to a control or a stress intervention (i.e., SECPT). The intervention was administered (at ~10 a.m.) in a testing room in the vicinity of the MRI scanner on average 30 min (range: 29–34) before the training on the MSL task (a self-initiated bimanual finger-tapping task, see next section), which took place in the MRI scanner. This timing was chosen, because SECPT-induced secretion of cortisol is known to reach peak levels after 25 min^45,46^. The MSL session was immediately followed by post-intervention/learning MRS (MRS post, Fig. 1). The post-intervention/learning MRS sequence thus started ~60 min after the start of the stress intervention.

To measure the time course of cortisol concentration, salivary samples were collected throughout the study (see Fig. 1 and below for details). At arrival (before baseline MRS measurements) and immediately before the control/stress intervention, vigilance was measured subjectively using the Stanford Sleepiness questionnaire and objectively using a Psychomotor Vigilance Task. A random serial reaction time task was administered before MSL (15 min after the intervention), in order to assess the effect of the intervention on general motor execution. Results with respect to the assessment of vigilance, general motor execution and sleep prior to the experimental session are reported in the Supplementary Results. The groups did not differ with respect to sleep characteristics, general motor performance and vigilance.

### Assessment of general motor execution

A random serial reaction time task^93^, implemented in Matlab Psychophysics Toolbox version 3, was used to assess general motor execution after the control/stress intervention but prior to MSL (Fig. 1). It is noteworthy that this task was performed while participants lied supine in the scanner but were not scanned. During this task, eight squares were presented on the screen, each corresponding to one of the eight keys on the specialized keyboard and to one of the eight fingers (no thumbs). The colour of the outline of the squares alternated between red and green, indicating rest and practice blocks, respectively. During the practice blocks, participants had to press as quickly as possible the key corresponding to the location of a green filled square that appeared on the screen. After a response, the next square changed to green (response-stimulus interval = 0 ms) following a random order. After 48 presses, the practice block automatically turned into a rest block and the outline of the squares changed from green to red. The task included four practice blocks, separated by 15 s rest intervals. Performance was measured in terms of speed (response time in ms) and accuracy (number of correct key presses).

### MSL task and behavioural measures

Participants were scanned while they were trained on a bimanual finger-tapping task implemented in Matlab Psychophysics Toolbox version 3. The task required participants to tap an eight-element finger sequence (eight fingers, no thumbs) on a specialized keyboard as rapidly and accurately as possible. The sequence to perform (4-7-3-8-6-2-5-1, where 1 and 8 correspond to the little fingers of the left and right hands, respectively) was presented on the screen during task practice. At the start of the training, a brief pre-training phase was included during which participants performed the sequence repeatedly and slowly until three consecutive correct sequences were completed. MSL consisted of 20 practice blocks and was followed by an immediate post test (after a 2 min break) of four practice blocks, in order to minimize the confounding effect of fatigue on end-training performance^57^. Each practice block was indicated with a green cross displayed in the middle of the screen with the sequence of numbers shown slightly above. Each practice block included 48 key presses (ideally corresponding to 6 correct sequences) after which the cross automatically turned red, indicating a rest block (duration 15 s). During the practice blocks, participants were instructed to continuously tap the sequence in a self-initiated manner until a stop signal (red cross) was given. During rest blocks, a sequence of eight asterisks (*-*-*-*-*-*-*-*) replaced the sequence of numbers and participants were instructed to keep their fingers still and look at the red fixation cross. Motor performance was measured in terms of speed (mean inter-response interval between two consecutive correct key presses in s) and accuracy (% of correct transitions). In addition, in order to assess the relationship between motor learning and measures of GABA (see MRS methods below), an online learning measure was computed as the percentage change in performance from blocks 1–2 to 19–20 of MSL. For both the training and the immediate post test, performance speed was analysed using a Block by Group RM ANOVA. In case of violation of the sphericity assumption, Greenhouse-Geisser corrections were applied. Online gains in performance speed were compared using an unpaired two-sample *t*-test (two-sided). Results related to the analysis of online gains are reported in the Supplementary Results (Section 2.4 and Fig. 3b).

### Stress induction method

In the stress condition, participants were exposed to a modified version of the SECPT^45,46,94^. The procedure is described in detail in Dolfen et al.^13,55^. The task required participants to immerse their feet in ice water (0–2 °C) as long as possible, while being videotaped for pretended analyses of facial expression and monitored by an unsociable and non-reinforcing experimenter. The duration of the cold-water stimulation was not provided to the participants; however, participants were instructed to withdraw their feet after 3 min. In contrast to the stress condition, participants in the control condition submerged their feet for 3 min in warm water (35–37 °C). They were neither monitored by an unsociable experimenter nor being videotaped.

With respect to subjective measures, participants were asked to rate their subjective feeling of stress, pain and unpleasantness on a visual analogue scale from 0 (‘Not at all’) to 100 (‘Very much’) immediately following the control/stress manipulation. Heart rate and blood pressure (systolic and diastolic) were assessed using an automatic upper arm blood pressure monitor (BP6000, Braun, Germany) before (pre), during and immediately following (post) feet immersion. Finally, for each participant, salivary cortisol samples were collected between the start of the baseline MRI session (~8.30 a.m.) and end of the post-intervention MRI session (~11.30 a.m.) using Salivette collection devices (Sarstedt Salivette) (see Fig. 1 for detailed timeline of cortisol measurements). After collection, the samples were stored at −20 °C until analysed, using immunoassay (analyses performed by Dresden Labservice GmbH, Germany). Subjective ratings were compared between groups using unpaired two-sample *t*-tests (two-sided). Physiological measures were analysed using Time by Group RM ANOVAs. In case of violation of the sphericity assumption, Greenhouse-Geisser corrections were applied. Results of planned pairwise comparisons were corrected using Bonferroni correction for multiple comparisons.

### Magnetic resonance imaging

Data were acquired with a Phillips Achieva 3T MRI system using a 32-channel head coil.

#### Magnetic resonance spectroscopy

MRS methods and quality measures are reported according to standards as outlined in the MRS‐Q guidelines^95^.

*MRS data acquisition*: For each MRS time point (baseline and post), MRS data were acquired from two separate voxels positioned over the left STR and left HC. Prior to each MRS time point, a (low-resolution) three-dimensionl (3D) T1-weighted structural image was acquired with a magnetization-prepared rapid-acquisition gradient echo (MP-RAGE) sequence (repetition time (TR)/echo time (TE) = 9.6/4.6 ms; voxel size = 1.2 × 1.2 × 2.0 mm^3^; field of view (FoV) = 250 × 250 × 222 mm^3^; 111 coronal slices). These lower-resolution anatomical scans were acquired in ~2 min and were used solely for the positioning of the MRS voxels. A single high-resolution T1-weighted 3D MP-RAGE sequence (TR = 9.5 ms, TE = 4.6 ms, inversion time  = 858.1 ms, FA = 9°, 160 slices, FoV = 250 × 250 mm^2^, matrix size = 256 × 256 × 160, voxel size = 0.98 × 0.98 × 1.20 mm^3^) was also obtained for each participant in a different experimental session (see Supplementary Fig. 1 for the full design). Using SPM12 (Welcome Department of Imaging Neuroscience, London, UK), the high-resolution T1-weighted image was independently co-registered to the two low-resolution images acquired in the baseline and post MRS time points, creating time point-specific high-resolution images used during further MRS data processing (see below).

MRS data were acquired from 40 × 25 × 25 and 30 × 30 × 30 mm^3^ voxels positioned over the HC and the STR, respectively, using the MEGA-PRESS sequence^96^ (14 ms sinc-Gaussian editing pulses applied at a frequency offset of 1.9 p.p.m. in the edit-ON experiment and 7.46 p.p.m. in the edit-OFF experiment, TR = 2 s, TE = 68 ms, 2 kHz spectral width, excitation water suppression). Three hundred and twenty and 224 averages were acquired for the HC and the STR, respectively, corresponding to total scan durations of 11 and 8 min. A higher number of averages were acquired for the HC voxel, to ensure sufficient signal-to-noise ratio^97^. An additional 16 water-unsuppressed averages were acquired from the same voxel and interleaved to allow for real-time frequency correction^98^, which is especially important after fMRI scanning^99^. With the exception of number of averages, and thus the total scan duration, these parameters were identical for the two MRS voxels. The HC voxel was centred on the left HC in the coronal view and positioned parallel to the long (antero-posterior) axis of the hippocampal body in the sagittal view. The STR voxel was centred over the left putamen. In the coronal and axial views, we checked that the voxel did not overlap with the ventricle and, as a consequence, only part of the caudate nucleus was covered. The order in which HC and STR voxels were acquired was counterbalanced across participants.

*MRS data analyses*: Data were analysed using the Gannet software 3.0 toolkit^100^. Individual spectra were frequency- and phase-corrected using spectral registration^101^. The GABA+ signal from the difference spectrum was modelled at 3.0 p.p.m. with a five-parameter Gaussian model, whereas the creatine and choline signals from the non-edited spectrum were fitted with a two-Lorentzian model. For each time point, GABA+ was measured as a ratio to total creatine within each voxel. It is noteworthy that the estimated GABA levels correspond to GABA+ macromolecules^102,103^ and are hence referred to as GABA+ levels in this manuscript. A GABA+ change measure was computed for use in correlational analyses with behaviour and functional activation by subtracting baseline GABA+ levels from post-intervention/learning levels (i.e., ΔGABA+, with a positive value indicating an increase in GABA+ levels).

Lipid contamination and water suppression were visually checked for each MRS time point. Quality of the MRS data was quantitatively assessed using GABA+ fit error, GABA+ signal-to-noise ratio and frequency drift (reflected by the SD of the frequency offset). The MRS voxels co-registered to the time point-specific, high-resolution anatomical images were segmented, to determine the different tissue fractions (grey matter, white matter (WM) and cerebrospinal fluid (CSF)) within each voxel and time point (see Supplementary Table 3). GABA+ values and quality metrics were analysed using Time (baseline vs. post) by Group RM ANOVAs.

To investigate the link between GABA+ and task performance, Spearman’s correlations were computed between GABA+ measures and online gain in performance speed during MSL. GABA+ measures of interest were baseline GABA+ and ΔGABA+. It is worth noting that as the GABA+ post measure was highly correlated to ΔGABA+ (*r* = 0.8), we opted to not include the GABA+ post variable in the correlation analyses, in order to limit the number of statistical tests. Correlations were computed within each group and next compared between groups using Fisher’s test. The Holm–Bonferroni procedure was used to correct for multiple correlations within each region of interest (i.e., four correlations/ROI). Results of these correlational analyses are reported in Supplementary Results Section 2.8. No links between behaviour and GABA measures were found.

#### Functional MRI data

*fMRI data acquisition*: BOLD signal during task practice was acquired with a T2* gradient echo-planar sequence using axial slice orientation that covers the whole brain (TR = 2000 ms, TE = 30 ms, FA = 90°, 54 transverse slices, 2.5 mm slice thickness, 0.2 mm interslice gap, FoV = 210 × 210 mm^2^, matrix size = 84 × 82 × 54 slices, voxel size = 2.5 × 2.56 × 2.5 mm^3^).

*fMRI preprocessing and data analyses*: Functional images were preprocessed and analysed using SPM12 implemented in Matlab (2020b). Preprocessing involved realignment to correct for motion, coregistration of functional and anatomical images, segmentation and spatial normalization to an average subject‐based template created using DARTEL in SPM12 (registered to the Montreal Neurological Institute (MNI) space). In order to form WM and CSF ROIs, the tissue maps (resulting from the segmentation of the anatomical image) were thresholded at a partial volume fraction of 0.99. WM masks were then eroded by two voxels in each direction. As CSF regions are typically small compared to WM regions, no erosion was applied^104^. The eroded WM and CSF masks were then used as noise ROIs. Finally, spatial smoothing was applied to the functional images (Gaussian kernel, 8 mm full-width at half-maximum [FWHM]).

The analysis of fMRI data was conducted in two serial steps accounting for fixed and random effects, respectively. Changes in brain responses were estimated using a general linear model including the responses to motor sequence practice and their linear modulation by performance speed (mean inter-response interval between correct consecutive key presses by block) during MSL practice. Performance speed, rather than accuracy, was chosen as a parametric modulator, because accuracy was not modulated by task practice (see Supplementary Results Section 2.5). The 15 s rest blocks occurring between each block of motor practice served as the baseline condition modelled implicitly in the block design. The regressors consisted of box cars convolved with the canonical hemodynamic response function. Movement errors (i.e., incorrect key presses) and key presses during rest were modelled as events of no interest. Movement parameters (derived from realignment of the functional volumes) and the average time series extracted from the noise ROIs were entered as regressors of no interest. High-pass filtering with a cut-off period of 128 s served to remove low-frequency drifts from the time series, and an autoregressive (order 1) plus white noise model and a restricted maximum likelihood algorithm was used to estimate serial correlations in fMRI signal. Subsequently, linear contrasts assessing the main effect of practice and its linear modulation by performance speed during MSL practice were generated. Modulation by speed contrasts identified regions where the magnitude of the responses increased (or decreased) as the interval between consecutive correct key presses decreased across practice. Similar to previous research (e.g., see refs. ^10^^,^^105–108^), the contrast images [SPM(CON)] were further spatially smoothed (Gaussian kernel 6 mm FWHM) and entered in second-level analysis accounting for inter-subject variance. It is noteworthy that the statistical model outlined above also included imaging data from the MSL retest session (see Supplementary Fig. S1). However, as retest data were outside the scope of our research questions, as no MRS data were acquired at retest, fMRI contrasts from the retest session were not considered in the second-level analyses described below.

In the second level, analyses were performed using full factorial ANOVAs. Results related to these analyses are reported in a previous paper^13^. To investigate the relationship between BOLD responses during MSL and GABA+ measures, we conducted ROI-based voxel-wise regression analyses. Specifically, we regressed the individual brain activation maps of (1) the main effect of practice and (2) the main effect of practice modulated by performance during training with the GABA+ measures of interest (baseline GABA+  and ΔGABA+). A final ANOVA was used to compare these relationships between groups. Statistical inferences were performed on a priori defined ROIs including bilateral hippocampi and bilateral STR (caudate nuclei and putamen) (as defined anatomically according to the AAL brain atlas)^109^. Analyses were performed using family-wise error correction for multiple comparisons over small volumes with a threshold of *p* < 0.05 (SVC^110,111^), followed by Holm–Bonferroni correction for multiple small volumes within each regression analysis (*p* < 0.05)^112^. For SVC, spheres (10 mm radius) were centred on coordinates from MSL literature in our ROIS [Hippocampal locations: (−18 −14 −28), (32 −36 −4)45 and (−22 −32 −12)^5^. Striatal locations: (20 12 2)^113^].

### Statistics and reproducibility

Statistical parametric mapping (SPM12; Welcome Department of Imaging Neuroscience, London, UK) was used for statistical analyses of BOLD data. All other data were analysed with SPSS Statistics 24 (IBM). Statistical tests in SPSS were performed by conducting Time by Group RM ANOVAs or unpaired two-sample *t*-tests. For RM ANOVAs, in case of violation of the sphericity assumption, Greenhouse-Geisser corrections were applied. Results of planned pairwise comparisons were corrected using Bonferroni correction for multiple comparisons. Sample size estimation was based on our previous work showing a significant correlation between MSL performance and cortisol response to stress^55^.

### Reporting summary

Further information on research design is available in the Nature Research Reporting Summary linked to this article.

## Supplementary information

Supplementary Material

Description of Additional Supplementary Files

Supplementary Data 1

Reporting Summary

## Data Availability

All source data underlying graphs in the main figures are available as Supplementary Data 1. The approval granted by the local ethics committee does not permit the sharing of individual data. Group-level data supporting our findings that are not available in the manuscript (including supplementary materials) are available from the corresponding author upon reasonable request.

## Code availability

The analyses in the manuscript are not based on custom algorithms or software. Scripts used for analysis in Matlab (version 2020b) and SPSS Statistics 24 (IBM) are available from the corresponding author upon reasonable request. Similarly, the scripts used for data collection (Matlab Psychophysics Toolbox version 3) are available upon request from the corresponding author.
