# Supplementary material for: A role for GABA in the modulation of striatal and hippocampal systems under stress
Source: Commun Biol. 2021 Sep 2;4:1033. doi: 10.1038/s42003-021-02535-x (PMC8413374; doi:10.1038/s42003-021-02535-x)
Supplement: Supplementary file 1 — Supplementary Material [file 42003_2021_2535_MOESM1_ESM.pdf]

1 Supplementary results

1.1 Sleep and vigilance

Sleep and vigilance scores are summarized in Supplementary Table 2. Average sleep duration for each night (3 nights before the experimental session) was estimated based on actigraphic data and the sleep diary. A 3 (Nights) x 3 (Groups: Control vs. stress cortisol responders (SCR) vs. stress cortisol non-responders (SCNR)) repeated measures (RM) ANOVA was conducted on average sleep duration. There was a main effect of night, whereby participants slept less during night 3 as compared to both nights 1 and 2 (both *ps* < .001) [main effect of night: $F_{(2,132)} = 69.207$, $\eta_p^2 = .465$, $p < .001$]. In the light of this effect, it's important to note that participants were required to be present in the lab for the experimental session between 8 and 9 am on the morning of day 4; and, importantly, they were instructed to wake up at least 1 hour before arrival (to account for the cortisol awakening response). It is highly likely that these instructions contributed to the reduced amount of sleep during night 3. It is worth noting, however that participants slept on average more than 7 hours per night. No main effect of group [$F_{(2,66)} = .750$, $\eta_p^2 = .011$, $p = .476$] nor a group by night interaction [$F_{(4,132)} = .207$, $\eta_p^2 = .007$, $p = .934$] were found. There were no group differences in subjective sleep quality of the night preceding the experimental session assessed with the St. Mary's Hospital questionnaire [one-way ANOVA with group (3) as between-subjects factor; $F_{(2,68)} = .291$, $p = .748$].

There were no differences in objective vigilance, assessed with the PVT, between time-points nor between groups [2 (Time: baseline vs. pre-intervention) x 3 (Groups: Control vs. SCR vs. SCNR) RM ANOVA on average reaction time; all $F \leq 1.247$, all $\eta_p^2 \leq .05$, all *ps* > .100] Similarly, no main or interaction effects were observed for subjective vigilance as assessed with the Stanford Sleepiness (SS) scale [2 (Time) x 3 (Groups) RM ANOVA on SS score; all Fs < 1, $\eta_p^2 \leq .002$, all ps > .1]. Altogether, these results indicate that vigilance was similar across time and groups.

1.2 Effectiveness of the stress intervention

Subjective ratings were analysed using one-way ANOVAs with group (Control vs. SCR vs. SCNR) as between-subjects factor. For all measures, the ANOVA yielded a main effect of

group [all Fs ≥ 59.979, all *p*s ≤ .001], whereby the intervention was rated significantly more stressful, painful and unpleasant by SCR and SCNR as compared to control participants [SCR vs. Control, all *p*s ≤ .001; SCNR vs. Control, all *p*s ≤ .001]. Post hoc tests showed that stress cortisol responders and non-responders did not statistically differ in their subjective ratings (SCR vs. SCNR, all *p*s ≥ .450).

To investigate the autonomic response, heart rate (HR; beats per minute), systolic (SBP) and diastolic (DBP) blood pressure (mmHg) were analysed using 3 (Time: pre vs. during vs. post intervention) x 3 (Groups) RM ANOVAs. Autonomic responses are depicted in Supplementary Figure 2A. For all measures, the analyses yielded a main effect of time [SBP: $F_{(1.54, 99.982)}$=18.642, η_p^2 = .237, p < .001; DBP: $F_{(1.779, 115.604)}$=21.440, η_p^2 = .248, p <.001; HR: $F_{(1.673,108.724)}$=80.006, η_p^2 = .552, p <.001], group as well as a time x group interaction [Group: SBP, $F_{(2,65)}$=13.758, η_p^2 = .305, p <.001; DBP, $F_{(2,65)}$= 7.898, η_p^2 = .196, p < .001; HR, $F_{(2,65)}$ = 5.844 , η_p^2 = .152, p = .005; Time x Group: SBP, $F_{(3.076, 99.982)}$ = 6.2; η_p^2 = .175, p < .001; DBP, $F_{(3.557, 115.604)}$=7.337; η_p^2 = .184, p < .001; HR, $F_{(3.345,108.724)}$ = 22.033, η_p^2 =.4, p < .001]. More specifically, blood pressure and heart rate were significantly increased during the intervention in both the SCR and SCNR groups as compared to the control group (SBP, SCR/SCNR vs. Control, *p* < .001, SCNR vs. SCR, *p* = 653; DBP, SCR/SCNR vs. Control, *p* < .001, SCNR vs. SCR, *p* = .693; HR, SCR/SCNR vs. Control, *p* < .001, SCR vs. SCNR, *p* = .789). With respect to HR, no group differences were observed pre- and post-intervention. Blood pressure post-intervention in SCNR was comparable to controls (SBP, *p* = .191; DBP, *p* = .564), while BP in SCR remained significantly elevated as compared to controls (SBP, *p* = .001, DBP, *p* < .001) but similar to SCNR (SCR vs. SCNR, SBP, *p* = .190; DBP, *p* = .400). Note that SBP in the SCR pre-intervention tended to be higher as compared to controls (SCR vs. Control, *p* = .069) but not different from SCNR (SCR v. SCNR, *p* = .525). The three groups did not differ with respect to DBP pre-intervention (all *p*s > .737). Overall, our data indicates that the two stress groups showed a significant and comparable autonomic stress response.

Lastly, the endocrine response was analysed with a 6 (Time) by 3 (Group) RM ANOVA. The time course of cortisol concentration for the different groups is depicted in Supplementary Figure 2B. The ANOVA yielded a main effect of time [$F_{(2.292, 146.717)}$ = 35.959, η_p^2 = .360, p < .001] and a time x group interaction [$F_{(4.585, 146.171)}$ = 8.987, η_p^2 = .22, p < .001]. There was no main effect of group [$F_{(2,64)}$ = .801, η_p^2 = .024, p = .453]. As expected, cortisol concentration was significantly higher in SCR as compared to SCNR and control groups at T25’

(SCR vs. SCNR/Control, both *p*s < .001) and T60' (SCR vs. SCNR/Control, both *p*s ≤ .006). No other differences between groups were observed (all other time-points, all *ps* ≥ .136).

Note that participants in the SCR group were selected based on the magnitude of the increase in cortisol from T0' to T25' (see Participants method section) and thus the group differences reported above at time-point T25' were expected. Supplementary Figure 2C presents the time course of cortisol concentration in the stress and control groups before the cortisol responder/non-responder classification. Stress and control groups were significantly different at T25' and T60'. Significant group differences at $p < .05$ based on pairwise comparisons (Bonferroni corrected) following a 6 (Time) x 2 (Groups: Control vs. Stress) RM ANOVA on cortisol [main effect of time and time x group interaction: both Fs ≥ 3.236, $\eta_p^2 \geq .045$, *ps* ≤.02; main effect of group: $F_{(1,69)} = .829$, $\eta_p^2 = .012$, $p = .366$].

1.3 Assessment general motor execution

A 4 (Blocks of practice) x 3 (Groups: Control vs. SCR vs. SCNR) RM ANOVA was performed on performance speed and accuracy from the random serial reaction time task. Due to computer malfunction, data from one control participant were missing. With respect to speed, the analysis yielded a main effect of block, indicating that participants got faster with practice [$F_{(2.257,146.730)} = 20.627$, $\eta_p^2 = .241$, $p < .001$]. Performance speed did not differ between groups [Group: $F_{(2,65)} = 1.207$, $\eta_p^2 = .036$, $p = .306$; Time x Group: $F_{(4.515,146.730)} = 1.225$, $\eta_p^2 = .036$, $p = .302$]. For accuracy, the analyses yielded no significant effects; accuracy was stable across blocks and similar between groups [all Fs ≤ 2.386, all $\eta_p^2 \leq .035$, all *ps* ≥ .092]. In summary, these results show that stress did not influence general motor performance.

1.4 MSL speed

A 20 (Blocks of practice during training) x 3 (Groups: Control vs. SCR vs. SCNR) RM ANOVA conducted on performance speed revealed a significant main effect of block [$F_{(4.884, 322.359)} = 79.883$, $\eta_p^2 = .55$, $p < .001$], indicating that speed improved with practice (Supplementary Fig. 3A). Performance improvement was comparable in all groups [Group: $F_{(2,66)} = 1.017$, $\eta_p^2 = .03$, $p = .367$; Block x Group interaction: $F_{(9.783, 322.359)} = .480$, $\eta_p^2 = .014$, $p = .899$]. As shown in Fig. 3A, SCR and SCNR showed very similar performance curves in contrast to the control group. It can be speculated that the autonomic component of the stress response had an impact on motor performance, potentially through increased

vigilance/arousal associated with the release of noradrenaline. As both the SCR and SCNR showed such a response, this would result in the similar performance curves as we see in the current study. A 4 (Blocks of practice during post-training test) x 3 (Groups) RM ANOVA indicated that performance speed further improved, reflected by a main effect of block [$F_{(3, 198)} = 4.602$, $\eta_p^2 = .065$, $p = .004$] during the immediate post-test and to a similar extent in all groups [Group: $F_{(2,66)} = .830$, $\eta_p^2 = .025$, $p = .44$; Block x Group: $F_{(6,198)}=1.023$, $\eta_p^2 = .03$, $p = .412$. As a measure of online learning, to be used in subsequent correlational analyses (see below), the percentage change in speed from blocks 1-2 to 19-20 of MSL was calculated. There were no group differences in the magnitude of online learning [one-way ANOVA; $F_{(2,68)} = .474$, $p = .625$] (Supplementary Fig. 3B). All groups showed significant gains in performance speed [one sample t-tests, all *ps* < .005].

1.5 MSL accuracy

A 20 (Blocks of practice during training) x 3 (Groups: Control vs. SCR vs. SCNR) RM ANOVA was conducted on performance accuracy. Performance accuracy remained stable with no significant group differences (Block: $F_{(11.658, 769.44)} = 1.349$, $\eta_p^2 = .020$, $p = .144$; Group: $F_{(1,51)} < .001$, $\eta_p^2 < .001$, $p = .998$; Block x Group: $F_{(23.316, 769.44)} = 1.251$, $\eta_p^2 = .037$, $p = .142$] (Supplementary Fig. 3A). A similar pattern was observed during the immediate post-test [Block: $F_{(3,198)} = .599$, $\eta_p^2 = .009$, $p = .616$; Group: $F_{(1,66)} = .886$, $\eta_p^2 = .026$, $p = .417$; Block x Group: $F_{(3, 198)}=1.025$, $\eta_p^2 = .03$, $p = .410$]. There were no group differences in the magnitude of online learning (computed as the percentage change in performance from blocks 1-2 to 19-20) [one-way ANOVA with 3 groups (Control vs. SCR vs. SCNR); $F_{(2,68)} = .474$, $p = .625$] (Supplementary Fig. 3A). However, while both the SCR and SCNR groups showed maintenance of accuracy [one sample t-tests, both *ps* ≥ .07], the control group showed a significant gain in accuracy [one sample t-test, $p = .019$].

1.6 Quality metrics related to MRS of GABA

Data quality metrics as well as MRS voxel tissue fractions for each MRS time-point and region of interest are detailed in Supplementary Table 3.

1.6.1 Striatum

Overall, the averaged quality and tissue fraction values are comparable to previous research with the exception of a slightly lower signal-to-noise ratio (SNR) in the current study

(averaged SNR = 15.34) as compared to previous studies (averaged reported SNR = 18-19; (1,2)). This lower SNR is likely due to the shorter scan duration in the current study (224 averages instead of 320 averages in previous studies, e.g. (1)) (3). There were no significant group differences nor any significant interactions between group and time for GABA fit error, GABA SNR or any of the tissue fractions (statistical results reported in Supplementary Table 3).

1.6.2 Hippocampus

Overall, tissue fractions are similar to what is reported in a previous study using a similar protocol by Huang et al. (4). There were no group differences or group x time-point effects in GABA fit error, GABA SNR or in any of the tissue fractions (statistics are reported in Table 3). The analysis did reveal a main effect of time on the SD of the water frequency offset ($F_{(1,58)}$= 9.663, p = .003). This was driven by a significantly larger SD post- as compared to baseline. This is likely caused by increased movement at the second timepoint due to the longer duration of the preceding scanning session. Importantly, the inclusion of SD of the frequency offset at baseline and post-intervention/learning as covariates did not influence the primary results reported in the main text (Time x Group RM ANCOVA on hippocampal GABA+ values; all $ps \geq .355$).

1.7 MRS of GABA+ in SCNR

GABA+ values for all groups are reported in Supplementary Table 4 and displayed in Supplementary Figure 4. A 2 (Time: baseline vs. post) x 3 (Groups: Control vs. SCR vs. SCNR) RM ANOVA on striatal GABA+ values yielded no effect of group or time, neither did we find a time x group interaction [Time: $F_{(1,65)}$ = .526, η_p^2 = .004, p = .471; Group: $F_{(2,65)}$ = .539, η_p^2 < .001, p = .586; Time x Group: $F_{(2,65)}$ = .202, η_p^2 < .003, p = .818]. Similarly, the 2 x 3 RM ANOVA on hippocampal GABA+ levels yielded no significant effects [Time: $F_{(1,58)}$ = .05, η_p^2 = .001, p = .825; Group: $F_{(2,58)}$ = .756, η_p^2 < .001, p = .474; Time x Group: $F_{(2,58)}$ = .806, η_p^2 < .027, p = .451].

1.8 Link between GABA+ and motor performance

For each region of interest, we used Spearman correlation to assess the relationship between GABA+ measures and online gain in performance speed during MSL. GABA+ measures of interest were baseline GABA+ and GABA+ change (i.e. ΔGABA+; computed as raw

change in GABA+ from baseline to post-intervention/learning). To assess if the stress, as compared to the control intervention, modulated the relationship between GABA+ and behaviour, correlations were computed within each group and next compared between groups using Fisher's test. The correlational analyses yielded no differences between groups as well as no significant within-group correlations between HC/STR GABA+ measures (baseline GABA+ or ΔGABA+) and performance gains (all *ps* > .1). Similarly, no correlations between HC/STR GABA+ measures and behaviour were observed across groups (all *ps* > .1).

2 Supplementary figures

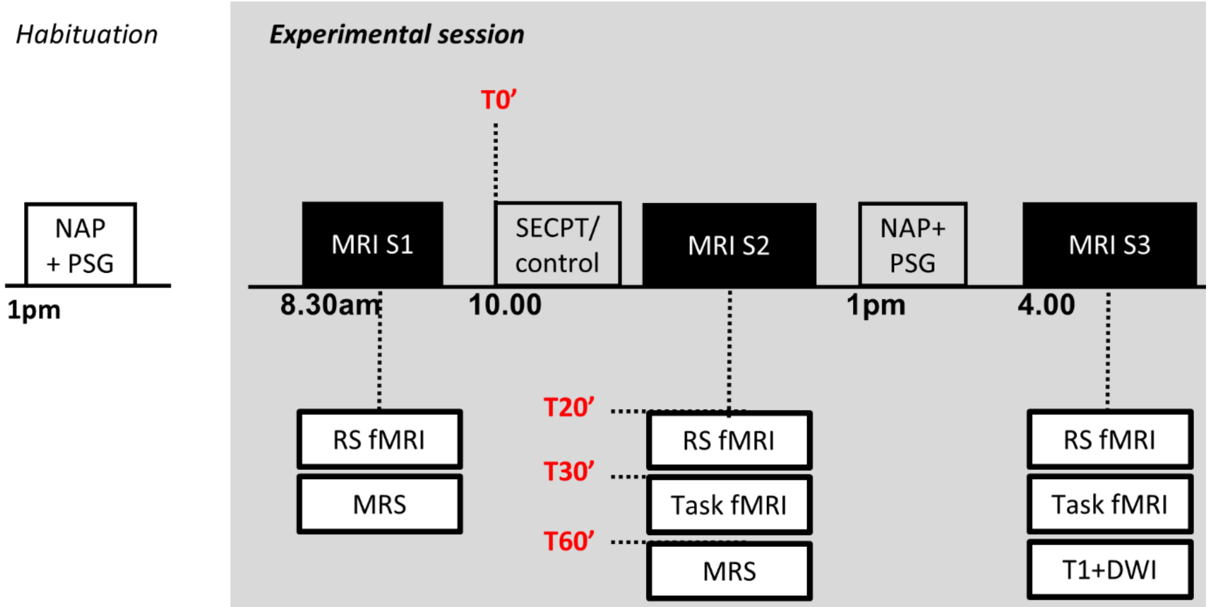

Supplementary Figure 1. One week prior to the experimental session, all participants completed a 90-min habituation nap (start ~1pm) in the sleep lab which was monitored using standard polysomnography (PSG, corresponding methods reported in (5)). On the day of the experimental session, each participant spent 9 consecutive hrs in the lab. Participants completed a total of 3 MRI sessions at 8.30am, 10am and 4pm, respectively referred to as MRI Sessions 1, 2 and 3. The first MRI session (8.30am – 9.15am) consisted of pre-intervention (baseline) measurements of (1) BOLD during resting state (RS) and (2) MR spectroscopy (MRS) (measure of GABA levels). At 10am, participants were subjected to the SECPT/control intervention (T0') outside the scanner and were immediately placed in the MRI scanner for post-intervention measurements (MRI session 2, 10.10am - 11.30am) that included (1) RS (T20'), (2) task-related fMRI during motor sequence learning (MSL) (T30') and (3) MRS (T60'). After this second MRI session, at 12pm, participants were offered a standardized lunch followed by a 90-min nap opportunity (starting ~1pm) that was recorded using PSG in the sleep lab. After this 90-min nap interval, at approximately 4pm participants were scanned again. The third MRI session (4pm – 5pm) included the acquisition of (1) RS fMRI, (2) task-related fMRI during a MSL Retest and (3) anatomical images (i.e. high resolution T1 and diffusion weighted imaging (DWI)).

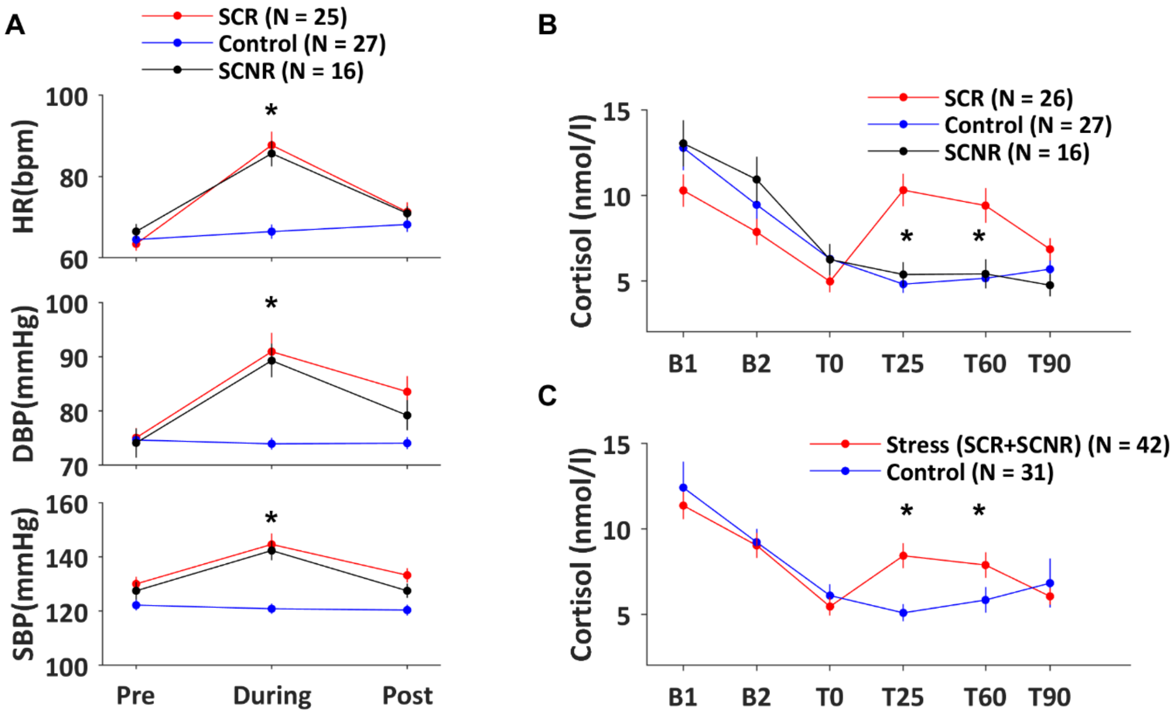

Supplementary Figure 2. Autonomic and endocrine stress responses (corresponding to Supplementary results section 2.2). **(A)** Heart rate (HR; beats per minute), systolic (SBP, mmHg) and diastolic blood pressure (DBP, mmHg) pre, during and post control/stress intervention. The SECPT resulted in a significant increase in HR and BP in both SCR and SCNR. **(B)** Time course of salivary cortisol concentration (nmol/L) for each group. **(C)** Time course of salivary cortisol concentration (nmol/L) in the control group *before exclusion* of control responders (N=3) and in the stress group across SCR and SCNR groups (see Participants methods section in main text). *represent significant group differences at $p < .05$. Cardiovascular data of one SCR were missing. Cortisol of two subjects at B1 (1 control, 1 SCR) and of one subject at T6H (control) were missing. B1 and B2 = Baseline 1 and 2. SCR = Stress Cortisol Responders. SCNR = Stress Cortisol Non-Responders. Error bars represent SEM.

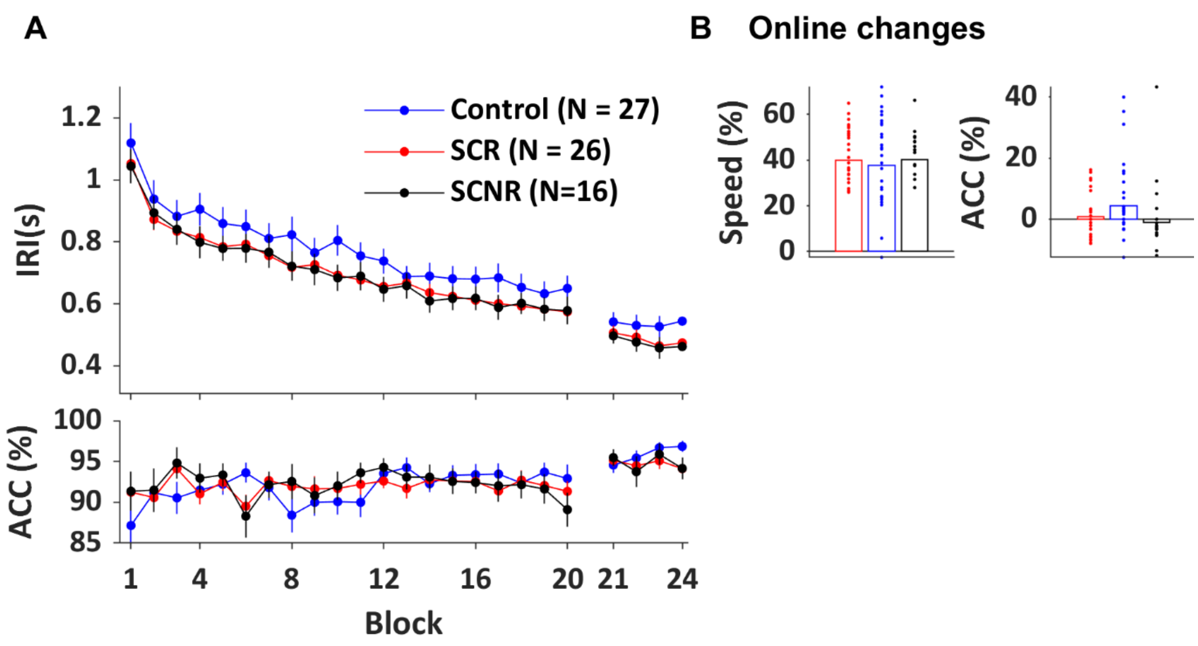

Supplementary Figure 3. Performance on the motor sequence learning (MSL) task. **(A)** Performance speed and accuracy plotted as a function of blocks of practice during MSL for the control, SCR and SCNR groups. While performance speed improved with practice in all groups, performance accuracy remained stable. **(B)** Online changes (% difference). Individual online changes plotted on top of group average. There were no group differences. Positive values reflect gains, negative values reflect deterioration in performance. SCR = Stress Cortisol Responders. SCNR = Stress Cortisol Non-Responders. ACC = accuracy. Error bars represent SEM.

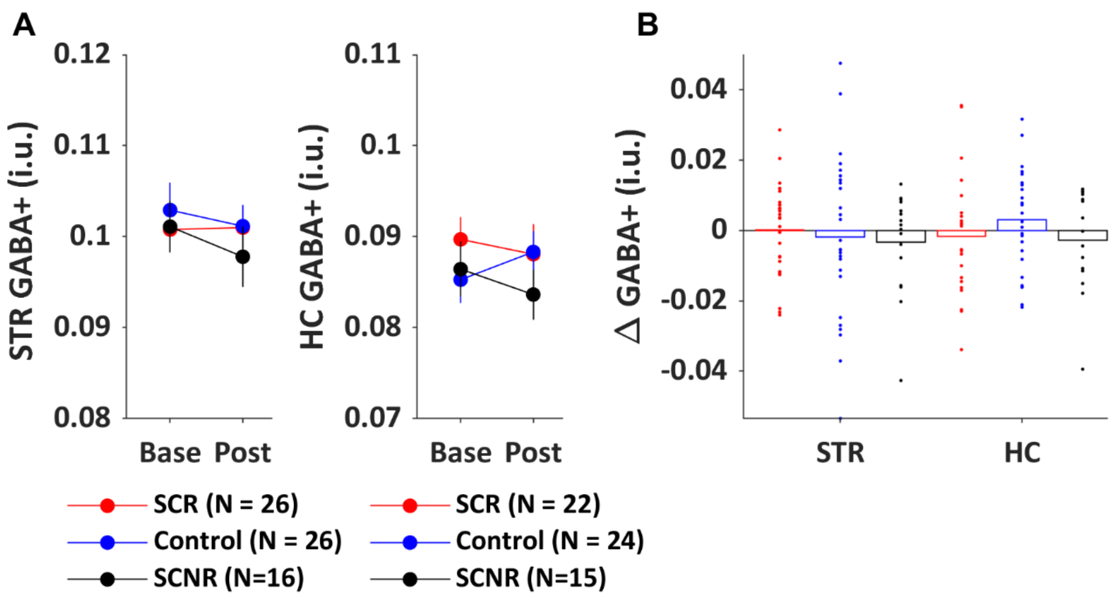

Supplementary Figure 4. (A) GABA+ levels for each time-point in the SCNR, SCR and control groups. Error bars reflect SEM. **(B)** Individual GABA+ changes (ΔGABA+) plotted on top of group average. Positive values reflect increases, negative values reflect decreases. STR = striatum. HC = hippocampus. Base = baseline pre-intervention. Post = post intervention/learning.

3 Supplementary Tables

Supplementary Table 1. Participant characteristics

	**Control**	**SCR**	**SCNR**	*Main effect of Group*
N	27	26	16	
Sex (F)	16	13	13	
Age (yrs)	22.88 ± 2.9	21.92 ± 2.36	22 ± 2.19	*p* = .842
Edinburgh Handedness	0.91 ± .14	0.89 ± .14	.92 ± .11	*p* = .700
Pain Catastrophizing Scale	10.41 ± 7.63	10.38 ± 6.59	8.44 ± 6.91	*p* = .627
Perceived Stress Scale	11.12 ± 6.34	12.12 ± 5.04	12.00 ± 5.44	*p* = .797
Epworth Sleepiness Scale	8.26 ± 3.27	7.96 ± 2.49	8.81 ± 3.29	*p* = .673
Beck Depression Scale	4.11 ± 3.52	4.46 ± 3.79	4.69 ± 7.06	*p* = .920
Beck Anxiety Scale	7.04 ± 6.5	6.54 ± 5.16	7.25 ± 6.56	*p* = .923
PSQI[a]	4	4	4	
Chronoscore (CRQ)*	54.41 ± 7.9	52.73 ± 7.73	51.63 ± 5.95	*p* = .470

Notes. Values are means ± standard deviation. PSQI = Pittsburgh Sleep Quality Index; CRQ = Circadian Rhythm Questionnaire. SCR = Stress Cortisol Responders. SCNR = Stress Cortisol Non-Responders. *p*-values are based on one way ANOVAs with Group (3) as between-subjects factor. [a]Median scores. * None of the participants were categorized as “Extreme morning” or “Extreme evening” type.

Supplementary Table 2. Sleep/vigilance scores

	**Control**	**SCR**	**SCNR**
N	27	26	16
**Sleep duration**			
Mean across the 4 nights	8h 11min ± 53min	8h 02min ± 32min	7h 51min ± 38min
Night 1	8h 45min ± 1h 9min	8h 37min ± 1h16min	8h 18min ± 38min
Night 2	8h 36min ± 1h 6min	8h 31min ± 56min	8h 3min ± 39min
Night 3	7h 11min ± 45min	7h 00min ± 36min	7h 14min ± 39min
**St. Mary's Sleep quality[a]**			
Night 3	4	4	4
**Psychomotor Vigilance Task (s)**			
At arrival	.373 ±.05	.358 ±.03	.347 ±.032
Pre-intervention	.364 ±.11	.36 ±.03	.354 ±.043
**Stanford sleepiness score**			
At arrival	2.5 ± .9	2.5 ±.8	2.4 ±.6
Pre-intervention	2.5 ± 1	2.6 ± .8	2.5 ± 1

Notes. Values are mean ± standard deviation. SCR = Stress Cortisol Responders. SCNR = Stress Cortisol Non-Responders. PVT data at arrival was missing for 3 participants (2 control, 1 SCNR) and pre-intervention for 1 participant (SCNR). [a]Median scores.

Supplementary Table 3. Data quality metrics for the tissue-corrected GABA+ levels and voxel tissue fractions

Group	N	Time	Data quality			Tissue fractions		
			**Fit Error (%)**	**GABA SNR**	**FreqStDevHz**	**GM**	**WM**	**CSF**
**Striatum**								
Control	26	Baseline	5.09 ± 1.93	14.48 ± 2.92	.60 ±.53	.58 ± .030	.36 ± .037	.060 ± .018
		Post	4.66 ± 1.68	15.07 ± 2.54	.79 ± .69	.58 ± .029	.35 ± .042	.063 ± .022
SCR	26	Baseline	4.06 ± 1.22	15.34 ± 2.58	.55 ±.22	.57 ± .025	.36± .031	.065 ± .025
		Post	4.41 ± 1.57	14.96 ± 3.16	.52 ± .16	.57 ± .034	.36 ± .035	.066 ± .025
SCNR	16	Baseline	4.06 ± 1.09	16.56 ± 2.89	.59 ±.26	.57± .024	.37 ± .037	.062 ± .020
		Post	4.31 ± 1.7	16.58 ± 3.1	.59 ±.28	.57± .029	.37 ± .040	.062 ± .019
Main effect of Time			$F_{(1,65)}$ = .062	$F_{(1,65)}$ = .044	$F_{(1,65)}$ = 1.712	$F_{(1,65)}$ = .076	$F_{(1,65)}$ = .358	$F_{(1,65)}$ = 2.081
			p = .805	p = .835	p = .195	p = .784	p = .552	p = .154
Main effect of Group			$F_{(2,65)}$ = 2.097	$F_{(2,65)}$ = 3.068	$F_{(2,65)}$ = 1.228	$F_{(2,65)}$ = .617	$F_{(2,65)}$ = .59	$F_{(2,65)}$ = .219
			p = .131	p = .053	p = .300	p = .543	p = .558	p = .804
Time x Group			$F_{(2,65)}$ =1.34	$F_{(2,65)}$ =.682	$F_{(2,65)}$ =2.729	$F_{(2,65)}$ =.737	$F_{(2,65)}$ =.514	$F_{(2,65)}$ =.613
			p = .269	p = .509	p = .073	p = .483	p = .600	p = .545
**Hippocampus**								
Control	24	Baseline	5.62 ± 1.43	10.85 ± 2.38	.65 ± .24	.57 ± .02	.36 ± .04	.07 ± .02
		Post	5.12 ± 1.02	11.86 ± 1.87	.82 ± .36	.56 ± .02	.37 ± .04	.07 ± .03
SCR	22	Baseline	5.06 ± 1.51	11.84 ± 2.41	.68 ± .22	.56 ± .03	.38 ± .02	.06 ± .02
		Post	5.44 ± 2.04	11.22 ± 2.55	.81 ± .35	.57 ± .04	.37 ± .02	.07 ± .02
SCNR	15	Baseline	4.61 ± 1.31	11.80 ± 1.82	.66 ± .23	.55 ± .02	.38 ± .03	.06 ± .02
		Post	5.29 ± 1.71	11.26 ± 2.28	.76 ± .40	.55 ± .02	.38 ± .03	.07 ± .02
Main effect of Time			$F_{(1,58)}$= .578	$F_{(1,58)}$= .026	$F_{(1,58)}$= 9.663	$F_{(1,58)}$= .068	$F_{(1,58)}$= .093,	$F_{(1,58)}$= .049
			p = .450	p = .872	p = .003	p =.795	p =.762	p = .825
Main effect of Group			$F_{(2,58)}$= .546	$F_{(2,58)}$= .064	$F_{(2,58)}$= .063	$F_{(2,58)}$= 1.104	$F_{(2,58)}$= 1.216	$F_{(2,58)}$= .469
			p = .582	p =.939	p = .939	p = .338	p = .304	p = .628
Time x Group			$F_{(2,58)}$= 2.187	$F_{(2,58)}$= 3.11	$F_{(2,58)}$= .232	$F_{(2,58)}$= .639	$F_{(2,58)}$= .636 ,	$F_{(2,58)}$= .312
			p = .121	p = .052	P = .794	p = .532	p = .533	p = .733

Notes. Values are means ± standard deviations. Statistics are based on a Time (2) x Group (3) RM ANOVA. Post = post-intervention/learning. SCR = Stress Cortisol Responders. SCNR = Stress Cortisol Non-Responders. SNR = signal to noise ratio, GABA = γ-aminobutyric acid, FreqStDevHz = standard deviation of the water frequency offset, GM = grey matter, WM = white matter, CSF = cerebrospinal fluid.

Supplementary Table 4. GABA+ values

**Striatum**				
		**Control (N = 26)**	**SCR (N = 26)**	**SCNR (N = 16)**
GABA + (i.u.)	Baseline	.103 ± .003	.101 ± .002	.102 ±.003
	Post	.101 ± .002	.100 ± .002	.098 ± 003
**Hippocampus**				
		**Control (N = 24)**	**SCR (N = 22)**	**SCNR (N = 15)**
GABA + (i.u.)	Baseline	.085 ± .013	.09 ± .011	.086 ± .011
	Post	.088 ± .011	.088 ± .015	.083 ± .010

Notes. Values are means ± standard deviations. All values are GABACr as provided in Gannet 3.0. SCR = Stress Cortisol Responders. SCNR = Stress Cortisol Non-Responders. i.u. = international units. Post = post-intervention/learning.

Supplementary Table 5. Functional imaging results for the MSL Training session across groups

**Area**	**X mm**	**Y mm**	**Z mm**	***p***
**Modulation by performance speed - regions wherein activity increases with practice**				
Right dorsal putamen	22	16	2	<.001
Left ventral putamen	-18	14	-4	<.001
Right ventral putamen	16	10	-8	<.001
Right caudate nucleus	18	-2	20	<.001

Notes. The significance threshold was set at $p_{corr} < .05$ (whole brain, FWE-corrected according to SPM12)

Supplementary Table 6. Neuroimaging results – within-group regression results

Region	X mm	Y mm	Z mm	# Voxels	T	p
**Main effect of practice**						
**Regression with STR baseline GABA+**						
**[+SCR]**						
Hippocampus	-16	-26	-10	215	3.38	.017
**Regression with HC baseline GABA+**						
**[-Control]**						
Hippocampus	28	-38	-2	7	2.84	.057
**Regression with ΔGABA+ STR**						
**[-SCR]**						
Hippocampus	-22	-16	-20	51	3.5	.013
**Regression with ΔGABA+ HC**						
No within-group suprathreshold clusters						
**Modulation by speed of performance**						
**Regression with STR baseline GABA+**						
No within-group suprathreshold clusters						
**Regression with HC baseline GABA+**						
No within-group suprathreshold clusters						
**Regression with ΔGABA+ STR**						
**[SCR]**						
Putamen (extending to caudate)	18	12	6	288	3.6	.010
	20	2	6		3.38	.017
**Regression with ΔGABA+ HC**						
No within-group suprathreshold clusters						

Notes: *p values are corrected for multiple comparisons (FWE) over small volumes. Coordinates for SVC are listed in the supplementary method section. Regressions with hippocampal GABA+: N Control = 23, N SCR = 22. Regressions with striatal GABA+: N Control = 26, N SCR = 26. ΔGABA+ = post-intervention/learning minus baseline. HC = Hippocampus. STR = Striatum. SCR = Stress Cortisol Responders.*

4 Supplementary References

1. Hermans L, Leunissen I, Pauwels L, Cuypers K, Peeters R, Puts NAJ, et al. Brain GABA levels are associated with inhibitory control deficits in older adults. 2018;38(36):0760–18.

2. Dharmadhikari S, Ma R, Yeh CL, Stock AK, Snyder S, Zauber SE, et al. Striatal and thalamic GABA level concentrations play differential roles for the modulation of response selection processes by proprioceptive information. 2015 Oct 5;120:36–42.

3. Mikkelsen M, Loo RS, Puts NAJ, Edden RAE, Harris AD. Designing GABA-edited magnetic resonance spectroscopy studies: Considerations of scan duration, signal-to-noise ratio and sample size. 2018;303:86–94.

4. Huang D, Liu D, Yin J, Qian T, Shrestha S, Ni H. Glutamate-glutamine and GABA in brain of normal aged and patients with cognitive impairment. 2017 Jul 1;27(7):2698–705.

5. Dolfen N, King BR, Schwabe L, Gann MA, Veldman MP, von Leupoldt A, et al. Stress Modulates the Balance between Hippocampal and Motor Networks during Motor Memory Processing. 2021 Jan 5;31(2):1365–82.
