# Supplementary material for: A role for GABA in the modulation of striatal and hippocampal systems under stress
Source: Commun Biol. 2021 Sep 2;4:1033. doi: 10.1038/s42003-021-02535-x (PMC8413374; doi:10.1038/s42003-021-02535-x)
Supplement: Supplementary file 2 — Description of Additional Supplementary Files [file 42003_2021_2535_MOESM2_ESM.pdf]

Description of Additional Supplementary files

File name: Supplementary Data 1

Description: Source data for Figures.
