# Supplementary material for: A role for GABA in the modulation of striatal and hippocampal systems under stress
Source: Commun Biol. 2021 Sep 2;4:1033. doi: 10.1038/s42003-021-02535-x (PMC8413374; doi:10.1038/s42003-021-02535-x)
Supplement: Supplementary file 4 — Reporting Summary [file 42003_2021_2535_MOESM4_ESM.pdf]

nature research

Corresponding author(s): Genevieve Albouy

Last updated by author(s): Jun 4, 2021

Reporting Summary

Nature Research wishes to improve the reproducibility of the work that we publish. This form provides structure for consistency and transparency in reporting. For further information on Nature Research policies, see our Editorial Policies and the Editorial Policy Checklist.

Statistics

For all statistical analyses, confirm that the following items are present in the figure legend, table legend, main text, or Methods section.

n/a	Confirmed	
☐	☒	The exact sample size (*n*) for each experimental group/condition, given as a discrete number and unit of measurement
☐	☒	A statement on whether measurements were taken from distinct samples or whether the same sample was measured repeatedly
☐	☒	The statistical test(s) used AND whether they are one- or two-sided<br>*Only common tests should be described solely by name; describe more complex techniques in the Methods section.*
☐	☒	A description of all covariates tested
☐	☒	A description of any assumptions or corrections, such as tests of normality and adjustment for multiple comparisons
☐	☒	A full description of the statistical parameters including central tendency (e.g. means) or other basic estimates (e.g. regression coefficient) AND variation (e.g. standard deviation) or associated estimates of uncertainty (e.g. confidence intervals)
☐	☒	For null hypothesis testing, the test statistic (e.g. *F*, *t*, *r*) with confidence intervals, effect sizes, degrees of freedom and *P* value noted<br>*Give P values as exact values whenever suitable.*
☒	☐	For Bayesian analysis, information on the choice of priors and Markov chain Monte Carlo settings
☒	☐	For hierarchical and complex designs, identification of the appropriate level for tests and full reporting of outcomes
☐	☒	Estimates of effect sizes (e.g. Cohen's *d*, Pearson's *r*), indicating how they were calculated

Our web collection on statistics for biologists contains articles on many of the points above.

Software and code

Policy information about availability of computer code

Data collection	The tasks were implemented in Matlab Psychophysics Toolbox version 3. The scripts used for data collection are available from the corresponding author on reasonable request.
Data analysis	Data processing was performed in Matlab (version 2020b), including SPM12 (Welcome Department of Imaging Neuroscience, London, UK) for BOLD data and Gannet software 3.0 toolkit (Edden et al., 2014) for MRS data. SPM was used for statistical analyses of BOLD data. All other data were statistically analysed with SPSS Statistics 24 (IBM). The analyses in the manuscript are not based on custom algorithms or software. Yet, the scripts used for analysis are available from the corresponding author on reasonable request

For manuscripts utilizing custom algorithms or software that are central to the research but not yet described in published literature, software must be made available to editors and reviewers. We strongly encourage code deposition in a community repository (e.g. GitHub). See the Nature Research guidelines for submitting code & software for further information.

Data

Policy information about availability of data

All manuscripts must include a data availability statement. This statement should provide the following information, where applicable:

- Accession codes, unique identifiers, or web links for publicly available datasets
- A list of figures that have associated raw data
- A description of any restrictions on data availability

The approval granted by the local ethics committee does not permit the sharing of individual data. Group-level data supporting our findings that are not already available in the manuscript (including supplementary materials) are available from the corresponding author upon reasonable request.

Field-specific reporting

Please select the one below that is the best fit for your research. If you are not sure, read the appropriate sections before making your selection.

☐ Life sciences ☒ Behavioural & social sciences ☐ Ecological, evolutionary & environmental sciences

For a reference copy of the document with all sections, see nature.com/documents/nr-reporting-summary-flat.pdf

Behavioural & social sciences study design

All studies must disclose on these points even when the disclosure is negative.

Study description	quantitative experimental (between-subjects design)
Research sample	Our research sample consisted of young healthy right-handed participants (age range: 18-30 years, N=80, 48 females). All participants were recruited in the area of Leuven, Belgium (see below for details on recruitment). Participants had no history of neurological or psychiatric diseases and were free of medications. Participants presented no signs of chronic pain, extreme stress, excessive daytime sleepiness or depression (see manuscript for details on the questionnaires used). All participants reported normal sleep quality and quantity during the month and the night prior to the study. We did not include extreme morning or evening chronotypes or shift-workers.
Sampling strategy	Participants were randomly sampled from the population of interest (see above). Sample size estimation was based on our previous work showing a significant correlation between offline gains in performance and cortisol response to stress (Dolfen et al. 2019). As earlier studies have shown that not all individuals show a cortisol response to the SECPT intervention (i.e. stress cortisol responders (SCR)) , individual cortisol data were analysed during collection. SCR are defined as participants with a stress-induced increase larger than 15.5% or 1.5nmol/l (Miller et al. 2013; classification used in Dolfen et al., 2019, 2021). Given the critical role of glucocorticoids in the impact of stress on learning and memory, data acquisition continued until the number of SCR (and control participants) reached the estimated sample size.
Data collection	Participants were blind to the study hypothesis during data collection.
Timing	Data collection started on 22/05/2018 and ended on 28/03/2020.
Data exclusions	Four participants in the control group were excluded because they were classified (using the criterion mentioned above) as cortisol responders. Two participants (one control and one SCR) were discarded because they were statistical outliers (average±3SDs) in performance speed and accuracy at the immediate post-training test. One control participant was excluded due to excessive motion during the fMRI training session (>2 voxels) and one participant (SCR group) was excluded because of a deviation from the experimental protocol. Three additional participants (one in each group) were excluded due to missing MRS data at one of the time-points. Accordingly, a total of 69 participants were included in the behavioural and stress physiology analyses (Control group (N = 27); Stress group (N = 42); see sampling strategy above for more information on sample sizes). In line with our previous work and given teh critical role of cortisol in the impact of stress on learning, the primary group comparison presented in the main text focused on the controls (N = 27) and cortisol responders in the stress group (SCR, N = 26; see sampling strategy for information on classification). For completeness, all results from the relatively small set of stress cortisol non-responders (N = 16) are detailed in the Supplementary Material. Additional participants were excluded from region-specific MRS analyses due to spectral artefacts at one of the time-points (hippocampus: N = 5) or due to extreme GABA+ levels (average + 3DS) (striatum: N=1, hippocampus: N=2). In the end, a total of 52 (control, N=26; SCR, N=26) and 46 (control, N=24; SCR, N=22) participants were included for striatum and hippocampus-specific MRS analyses.
Non-participation	No participants dropped out/declined participation.
Randomization	Participants were randomly allocated to one of two groups, i.e. control or stress group.

Reporting for specific materials, systems and methods

We require information from authors about some types of materials, experimental systems and methods used in many studies. Here, indicate whether each material, system or method listed is relevant to your study. If you are not sure if a list item applies to your research, read the appropriate section before selecting a response.

Materials & experimental systems

n/a	Involved in the study
☒	☐ Antibodies
☒	☐ Eukaryotic cell lines
☒	☐ Palaeontology and archaeology
☒	☐ Animals and other organisms
☐	☒ Human research participants
☒	☐ Clinical data
☒	☐ Dual use research of concern

Methods

n/a	Involved in the study
☒	☐ ChIP-seq
☒	☐ Flow cytometry
☐	☒ MRI-based neuroimaging

Human research participants

Policy information about studies involving human research participants

Population characteristics: See above

Recruitment: Participants were recruited via advertisements posted on student job websites or shared on social media.

Ethics oversight: Medical Ethics Committee University Hospital Leuven, Belgium; B322201525025

Note that full information on the approval of the study protocol must also be provided in the manuscript.

Magnetic resonance imaging

Experimental design

Design type: Task-related functional MRI: block design

Design specifications: Participants were scanned while they were trained on a bimanual finger-tapping task. The fMRI session consisted of 20 practice blocks (referred to as training in the behavioral performance measures section below) and was followed by an immediate post-test of 4 practice blocks. Practice blocks were alternated with 15 s rest-intervals. The 15-second rest blocks occurring between each block of motor practice served as the baseline condition modelled implicitly in the block design. Each practice block consisted of 48 keypresses after which it automatically turned into a rest block. Accordingly there was no fixed block duration: it decreased as participants got faster on the task.

Behavioral performance measures: Performance during fMRI was measured in terms of speed (mean inter-response interval between two consecutive correct keypresses in s) and accuracy (% of correct transitions). For both the training and the post-test, performance speed was analysed using a Block by Group repeated measures ANOVA. In case of violation of the sphericity assumption, Greenhouse-Geisser corrections were applied.

Acquisition

Imaging type(s): anatomical (T1-weighted image); functional fMRI; MR Spectroscopy of GABA

Field strength: 3

Sequence & imaging parameters: Anatomical data was acquired using a high-resolution T1-weighted 3D MP-RAGE sequence (TR = 9.5 ms, TE = 4.6 ms, TI = 858.1 ms, FA = 9°, 160 slices, FoV = 250 × 250 mm2, matrix size = 256 × 256 × 160, voxel size = 0.98 × 0.98 × 1.20 mm3). MRS data were acquired from 40x25x25 and 30x30x30mm3 voxels positioned over the hippocampus and the striatum, respectively, using the Mescher–Garwood point resolved spectroscopy (MEGA-PRESS) sequence ([89]) (14 ms sinc-Gaussian editing pulses applied at a frequency offset of 1.9 ppm in the edit-ON experiment and 7.46 ppm in the edit-OFF experiment, TR = 2 seconds, TE= 68 ms, 2 kHz spectral width, excitation water suppression). fMRI data during task practice was acquired with a T2* gradient echo-planar sequence using axial slice orientation that covers the whole brain (TR =2000 ms, TE=30 ms, FA= 90°, 54 transverse slices, 3 mm slice thickness, 0.2 mm inter-slice gap, FoV = 210 × 210 mm2, matrix size = 84 × 82 × 54 slices, voxel size = 2.5 × 2.56 × 2.5 mm3).

Area of acquisition: fMRI and anatomical: whole brain scan; MRS: ROI, acquisition box centered over either the left hippocampus or left striatum. The HC voxel was centered on the left hippocampus in the coronal view and positioned parallel to the long (antero-posterior) axis of the hippocampal body in the sagittal view. The STR voxel was centered over the left putamen. In the coronal and axial views, we checked that the voxel did not overlap with the ventricle, and, as a consequence, only part of the caudate nucleus was covered

Diffusion MRI ☐ Used ☒ Not used

Preprocessing

Preprocessing software: Functional images were preprocessed and analyzed using SPM12 implemented in Matlab (2020b). Preprocessing included the realignment of the functional time series, segmentation of the structural T1-image, coregistration of functional images to the

	structural T1-image, spatial normalization (see below) and spatial smoothing (Gaussian kernel, 8 mm full-width at half-maximum [FWHM]).
Normalization	Spatial normalization was performed on both functional and anatomical images using non-linear registration with the DARTEL tool in SPM12 that makes use of individual flow fields.
Normalization template	Average subject-based template created using DARTEL in SPM12 (registered to the MNI space)
Noise and artifact removal	Average time series extracted from the cerebrospinal fluid and white matter segments were entered as regressors of no interest in first-level fixed effects GLM (see below)
Volume censoring	No censoring was applied

Statistical modeling & inference

Model type and settings	We used a mass-univariate approach for the analysis of the fMRI data. The analysis was conducted in 2 serial steps accounting for fixed and random effects, respectively. At the first level, changes in brain responses were estimated using a general linear model including the responses to task practice and their linear modulation by performance speed during task practice. The 15-second rest blocks occurring between each block of motor practice served as the baseline condition modelled implicitly in the block design. Regressors of interest consisted of box cars convolved with the canonical hemodynamic response function. Movement parameters (derived from realignment of the functional volumes) as well as the average time series extracted from the cerebrospinal fluid and white matter segments were entered as regressors of no interest. High-pass filtering with a cut-off period of 128 s served to remove low-frequency drifts from the time series and an autoregressive (order 1) plus white noise model and a restricted maximum likelihood (ReML) algorithm was used to estimate serial correlations in fMRI signal. The second level analyses were performed using full factorial ANOVAs (see below).
Effect(s) tested	We tested whether acute stress modulated the relationship between BOLD responses during task practice and GABA measures. To test this we conducted whole-brain voxel-wise regression analyses. A final ANOVA was used to compare these relationships between groups.

Specify type of analysis: ☐ Whole brain ☒ ROI-based ☐ Both

Anatomical location(s)	Statistical inferences were performed on a priori defined regions of interest including bilateral hippocampi as well as bilateral striatum (caudate nuclei and putamen) as defined anatomically according to the AAL brain atlas (Tzourio-Mazoyer et al., 2005).

Statistic type for inference (See Eklund et al. 2016)	voxel-wise
Correction	Analyses were performed using family-wise error (FWE) correction for multiple comparisons over small volume within the ROIS with a threshold of $p < 0.05$ (SVC; Poldrack et al., 2007), followed by Holm-Bonferroni correction for multiple ROIs within each regression analysis ($p < 0.05$) (Holm, 1979). For SVC, spheres (10 mm radius) were centered on coordinates from literature in our ROIS.

Models & analysis

n/a	Involved in the study
☒	☐ Functional and/or effective connectivity
☒	☐ Graph analysis
☒	☐ Multivariate modeling or predictive analysis
